# Combined Effect of Salt Stress and Nitrogen Level on the Primary Metabolism of Two Contrasting Hydroponically Grown *Cichorium spinosum* L. Ecotypes

**DOI:** 10.3390/biom13040607

**Published:** 2023-03-28

**Authors:** Martina Chatzigianni, Dimitrios Savvas, Evgenia-Anna Papadopoulou, Konstantinos A. Aliferis, Georgia Ntatsi

**Affiliations:** 1 Laboratory of Vegetable Production, Department of Crop Science, Agricultural University of Athens, 75 Iera Odos, 11855 Athens, Greece; 2 Laboratory of Pesticide Science, Department of Crop Science, Agricultural University of Athens, 75 Iera Odos, 11855 Athens, Greece; 3 Department of Plant Science, Macdonald Campus, McGill University, Ste-Anne-de-Bellevue, QC H9X 3V9, Canada

**Keywords:** abiotic stress, ecotypes, metabolism regulation, nitrogen, proline, salt stress, *stamnagathi*

## Abstract

*Stamnagathi* (*Cichorium spinosum* L.) is an indigenous plant species well-known for its health-promoting properties. Salinity is a long-term issue with devastating consequences on land and farmers. Nitrogen (N) constitutes a crucial element for plant growth and development (chlorophyll, primary metabolites, etc.). Thus, it is of paramount importance to investigate the impact of salinity and N supply on plants’ metabolism. Within this context, a study was conducted aiming to assess the impact of salinity and N stress on the primary metabolism of two contrasting ecotypes of *stamnagathi* (montane and seaside). Both ecotypes were exposed to three different salinity levels (0.3 mM—non-saline treatment, 20 mM—medium, and 40 mM—high salinity level) combined with two different total-N supply levels: a low-N at 4 mM and a high-N at 16 mM, respectively. The differences between the two ecotypes revealed the variable responses of the plant under the applied treatments. Fluctuations were observed at the level of TCA cycle intermediates (fumarate, malate, and succinate) of the montane ecotype, while the seaside ecotype was not affected. In addition, the results showed that proline (Pro) levels increased in both ecotypes grown under a low N-supply and high salt stress, while other osmoprotectant metabolites such as γ-aminobutyric acid (GABA) exhibited variable responses under the different N supply levels. Fatty acids such as *α*-linolenate and linoleate also displayed variable fluctuations following plant treatments. The carbohydrate content of the plants, as indicated by the levels of glucose, fructose, *α*,*α*-trehalose, and myo-inositol, was significantly affected by the applied treatments. These findings suggest that the different adaptation mechanisms among the two contrasting ecotypes could be strongly correlated with the observed changes in their primary metabolism. This study also suggests that the seaside ecotype may have developed unique adaptation mechanisms to cope with high N supply and salinity stress, making it a promising candidate for future breeding programs aimed at developing stress tolerant varieties of *C. spinosum* L.

## 1. Introduction

Salinity, drought, heat, and cold are among the major abiotic stresses that negatively impact plant growth, biomass production, productivity, seed production, and fruit nutritional characteristics while causing metabolic changes in the most widely used (commercial) vegetables [1]. Moreover, extreme weather conditions can lead to salinity issues in soil, either due to a high percentage of surface evaporation or adequate precipitation. The productivity of agricultural lands can be further reduced by the use of poor irrigation water quality [2,3,4]. The development of salinity stress symptoms in plants depends on various factors including their genotype, growth stage, and the duration and intensity of the stress imposed [5,6]. Following plant exposure to salinity stress, photosynthesis is inhibited due to stomatal closure, which can be caused either by a decline in leaf turgor or the production of related hormones in both shoots and roots [7,8]. However, a low photosynthetic rate may also be the result of increased Na^+^ levels, which are responsible for the inhibition of enzymatic activity related to photosynthesis, and consequently reduce the leaf chlorophyll content [9,10]. Salinity also affects plants’ primary metabolism through changes in the levels of various metabolites involved in osmoprotection, energy production, hormone alteration, and stress responses. Indeed, nutrient imbalance leads to Na^+^ and Cl^−^ antagonism with essential macronutrients for plants’ development and yield macronutrients, such as Ca^2+^, Mg^2+^, K^+^, NH_4_^+^, and NO_3_^−^ [11,12]. Furthermore, reduced micronutrient levels seem to be related to high pH values in the soil under salt stress conditions [13]. To counteract such stress, plants have developed complex molecular, biochemical, and physiological growth and development mechanisms [14,15,16,17].

Soil salinity causes either osmotic stress, resulting from reduced water absorption, or plant toxicity due to high salt concentration [18,19,20,21,22,23,24,25]. High salt levels in the roots have a detrimental effect on cells’ growth and metabolism. Furthermore, nutrient starvation and oxidative stress can be observed due to a high concentration of salts, which requires more time to accumulate inside the plants and therefore results in a posterior ionic effect [26]. The dissection of plant behavior under major stresses helps towards understanding the metabolic changes they undergo in order to combat stress. Such an approach could lead to the introduction of new strategies towards improving tolerance, rather than relying solely on the selection of tolerant species [27]. The mechanisms that plants have developed to overcome salinity stress include signal transduction and the activation of several stress-related metabolites and genes. Another mechanism that plants employ to avoid the negative impact of increasing salt concentration is the blockage of Na^+^ from the shoots [23,28]. Moreover, tissue tolerance obtained via K^+^ retention in the cytosol and the intracellular compartmentalization of Na^+^ in the vacuole can be considered a mechanism of tolerance to this stress. Such mechanisms are critical to increase cell osmotic pressure and decrease Na^+^ toxicity, which is associated with the tendency of Na^+^ to be replaced by K^+^ in key enzymes of the cytosol and organelles [29,30].

Plants exhibit variable responses to stresses. However, even if the physiological mechanisms are species- and developmental-stage dependent, the main cellular processes are common in most plant species [3]. In addition, different stimuli can cause osmotic and oxidative stresses and denaturation of proteins, changes that may lead to plants accumulating compounds known as “compatible solutes” and the activation of stress-induced proteins, as well as the triggering of the reactive oxygen species (ROS) scavenging systems [16,17]. These compatible solutes are compounds of high soluble capacity and low molecular weight, exhibiting no toxic effects when accumulated in plants. Furthermore, a large number of these compatible solutes protect plants from cell dehydration, thus being referred to as osmoprotectants. Among these are polyols, Pro, trehalose, sucrose, and quaternary ammonium compounds (QACs), i.e., alanine betaine, glycine betaine, proline betaine, hydroxyproline betaine, choline O-sulfate, and pipecolate betaine [31]. Proline (Pro) is a metabolite that protects plants from the detrimental consequences of their exposure to high levels of salinity. It not only constitutes a compatible osmolyte but also serves as an enzyme protectant, a cell redox balancer, a free radical scavenger, a cytosolic pH buffer, and a stabilizer of subcellular structures [32,33], contributing to plant’s salinity tolerance. Previous studies have indicated that high Pro concentrations in the shoot parts of plants grown under high salinity were due to either the expression of genes encoding enzymes of Pro synthesis or decreases in the activity of proline oxidation enzymes [34]. Pro also plays a major role in the protection of the photosynthetic activity of *Opuntia streptacantha* plants under salinity stress [35] and has a crucial and protective role against NaCl-induced cell death via decreases in the level of lipid peroxidation and ROS [36], as well as the improvement of membrane integrity via the increased expression of antioxidant genes.

It is well documented that salinity stress causes metabolic and nutrient imbalances, which together result in a complex physiological syndrome [37]. Several plant metabolomics studies have already indicated that various metabolites are strongly correlated to salinity stress, including amino acids (AAs), sugars, polyols, and Krebs cycle intermediates, which act as biochemical targets under stress conditions [6,38]. Recent studies clearly indicated that sugars, such as sucrose (Suc), glucose (Glu), and fructose (Fru), along with Pro, citrate, malate, and succinate, were substantially higher in the halophyte *Thellungiella halophila* compared to *Arabidopsis thaliana*. Moreover, raffinose-pathway metabolites, such as raffinose, myo-inositol, and galactinol, exhibited a greater accumulation in *T. halophila* than in *A. thaliana* under salinity conditions. In contrast, the levels of malate, fumarate, aspartate, and phosphate decreased in the halophyte [39,40].

When plants are exposed to high levels of salinity, they often experience reduced N uptake and utilization. This is because high levels of salt in the soil can disrupt the uptake of water and nutrients, including N. N is an essential nutrient for plants, playing a crucial role in their growth and development. It is a key component of AAs, nucleic acids, chlorophyll, and many other essential molecules in plants [41]. Therefore, it is important to consider both N and salinity levels when managing plant growth and development, especially in environments with high salinity levels. Adequate N fertilization can help mitigate the negative effects of salinity on plant growth while also promoting healthy development and maximizing yields [42]. *Stamnagathi* (*Cichorium spinosum* L.) is a widespread and well-known wild chicory species in Crete and belongs to the Asteraceae family. Its flower-heads range from blue to pale violet and sometimes white, and they bloom from May to July–August. This dwarf perennial plant can undoubtedly be considered as a “super food”. This is attributed to its diuretic, purgative, antiseptic and anti-rheumatic properties, and its high content in antioxidants, Vitamins E and K1, [43], *ω*-3 fatty acids, and mineral elements [44]. *Stamnagathi* can be found in both mountainous and coastal areas in the Mediterranean basin, [45] indicating its potential tolerance to several abiotic stresses (N limitation, salinity, drought, etc.) [46,47]. *Stamnagathi* is a leafy green and as such is selected for high growth rates that rely on high N supply rates. In general, the suggested range of N concentrations in nutrient solutions used for hydroponic cultivation of other vegetable ranges from 10 to 16 mmol L^−1^ [46].

Taking into consideration the abovementioned, in this study, the impact of NaCl concentration and N level in the supplied nutrient solution on the primary metabolism of *stamnagathi* was investigated. For this reason, two contrasting *stamnagathi* ecotypes were grown hydroponically and exposed to two different total-N supply levels, a low-N level at 4 mM and a high-N level at 16 mM, in combination with three different salinity levels (0.3 mM—non-saline treatment, 20 mM—medium, and 40 mM—high salinity level). The two ecotypes of this study were grown in a completely different environment. Specifically, one of the ecotypes was collected from the mountain Lefka Ori (1200 m altitude) in the Prefecture of Chania, characterized by poor vegetation and a low organic matter content, and consequently by low soil N concentrations. Therefore, we hypothesized that this ecotype could sustain its grown even when grown under a low N supply compared to the other ecotype. On the other hand, the second ecotype was collected from the coastal area of Stavros, a site located in Akrotiri in northeastern Crete. Therefore, we hypothesized that this ecotype could tolerate high salinity levels in the nutrient solution compared to the montane ecotype.

## 2. Materials and Methods

### 2.1. Plant Material

*Cichorium spinosum* L. seeds, originating from two different Cretan regions (local landraces), were collected with the aim of investigating the effect of salinity and N-supply level on their metabolism. More specifically, seeds of the montane (Tavri, Lefka Ori, Chania) and the seaside (Stavros, Akrotiri Chania) ecotype were collected and transferred to the Seed Bank of the Mediterranean Institute of Chania (MAICh). Afterwards, they were incubated in Petri plates at 20 °C under a 12-h photoperiod until reaching the “cotyledon” stage. Prior to transplantation, the germinated seeds were transferred to 84-hole trays, using a mixture of peat and perlite (3:1, *v*/*v*) as a substrate, and grown in an unheated greenhouse. Perlite bags (Perloflor Hydro 1, 33 L) were employed for seedling transplanting, and plants were cultivated in an open hydroponic system in MAICh’s glasshouse with a North–South orientation, located at 35°29′40.32″ N latitude and 24°02′57.51″ E longitude.

### 2.2. Growth Conditions and Experimental Design

In this study, a factorial combination resulted in twelve treatments: two *stamnagathi* ecotypes from either the montane (M) or the coastal (C or S-seaside) site, three NaCl concentrations (0.3, 20, or 40 mM), and two levels of total-N concentrations (4 or 16 mM). A randomized complete-block design with four replicates per treatment was applied, resulting in 48 experimental units (plots) with 12 plants (3 bags × 4 plants per bag) in each plot (*n* = 576 plants). After transplanting, the plants were subjected to the salinity and N treatments. The nutrient solutions used in this study were prepared according to Savvas and Adamidis [48]. The concentrations of the macro-and micronutrients were applied according to our previous study [47]. The pH of the nutrient solution was adjusted to 5.6. The electrical conductivity (EC) values ranged between 2.10, 4.10, and 6.10 dS m^−1^ for the three different salinity levels, 0.3 mM non-salt control, 20 mM, and 40 mM NaCl, respectively, during the whole cropping period. Two days before transplanting, the perlite bags were irrigated with the nutrient solution until saturation. After 24 h, two holes were made at the bottom of the bags to collect the drainage solution. Plants were drip irrigated daily with the nutrient solution using pumps connected to an electronic timer, supplying 35 mL min^−1^ to each plant. Transplanting occurred on 30 January, with an irrigation program of four irrigations per day, adjusted according to the climate conditions and drainage solution. Two harvests were performed 60 and 118 days following transplantation, respectively, by cutting the main stem with sharp knives. No plant protection measures or heating was implemented throughout the cropping period.

### 2.3. Gas Chromatography-Electron Impact-Mass Spectrometry (GC/EI/MS) Metabolomics Analysis of Cichorium spinosum L. Leaves

The fresh *stamnagathi* leaves were collected from all experimental units during the second harvest for metabolomics analysis. For each treatment, ten healthy, fully expanded leaves were randomly collected from three plants and pooled, resulting in a total of four pooled samples per treatment. The leaves were collected, placed in falcon tubes (50 mL), and their metabolism was quenched by liquid N_2_. Samples were pulverized in a mortar, using a pestle under liquid N_2_, and were stored at −80 °C until further processing. A portion of the pulverized leaf tissues (25 mg) was transferred into Eppendorf tubes (2 mL) and 1 mL of methanol:ethyl acetate (50:50, *v*/*v*) (GC/MS grade, 99.9% purity, Carlo Erba Reagents, val de Reuil, France) was added for metabolite extraction. The resulting suspensions were spiked with ribitol (20 mL, 0.2 mg per mL of methanol) (99.8%, *v*/*v*, Sigma-Aldrich Ltd., Steinheim, Germany), which served as the internal standard (IS). A previously described extraction and derivatization protocol was employed [49,50], which includes sonication, agitation, filtering, and finally evaporation of the extracts. Derivatization of the dry extracts was performed following a two-step process by initially adding 80 μL of methoxylamine hydrochloride (solution in pyridine, 20 mg mL^−1^) (98%, *w*/*w*, Macherey and Nagel, Düren, Germany) and then 80 μL of N-Trimethylsilyl-N-methyl trifluoroacetamide (MSTFA, Macherey and Nagel, Düren, Germany).

An Agilent 6890N gas chromatography-electron impact-mass spectrometry GC/EI/MS platform (Agilent Technologies Inc., Santa Clara, CA, USA), equipped with the 5973 inert mass selective detector (MSD) and the 7683 autosampler, was used for the analyses, applying previously described settings [49,50]. Samples (1 μL) were injected onto a column (HP-5MS capillary column, 30 m, i.d. 0.25 mm, film thickness 0.25 μm, Agilent Technologies Inc., Santa Clara, CA, USA) applying a split ratio of 5:1. The obtained total ion chromatograms were deconvoluted using the software AMDIS v.2.66 (NIST; Gaithersburg, MD, USA) and the MS database of the National Institute of Standards and Technology, NIST ’08 (NIST; Gaithersburg, MD, USA). Additionally, analytical standards of selected plants’ metabolites were analyzed for the absolute annotations (Sigma-Aldrich Ltd., Steinheim, Germany).

Data pre-processing was performed using the software MS-Dial v.3.70 [49,51] and the mining for the discovery of trends and biomarkers was performed using the bioinformatics software SIMCA-P v.13.0.3 (Umetrics, Sartorius Stedim Data Analytics AB, Umeå, Sweden) following a previously described pipeline [49,50]. Orthogonal partial least squares-discriminant analysis (OPLS-DA) was employed for the discovery of trends and the biomarkers of plants’ stresses. The supervised OPLS-DA modeling of the data is easy to interpret and provides information on the variables with the highest discriminatory power. It also provides information on the variance of X that is related to the groups and the systematic information that is unrelated to the observed discriminations. The metabolite profiles of the plants from all treatments were used to develop such a model.

### 2.4. Experimental and Bioanalytical Protocols

**Figure 1:**
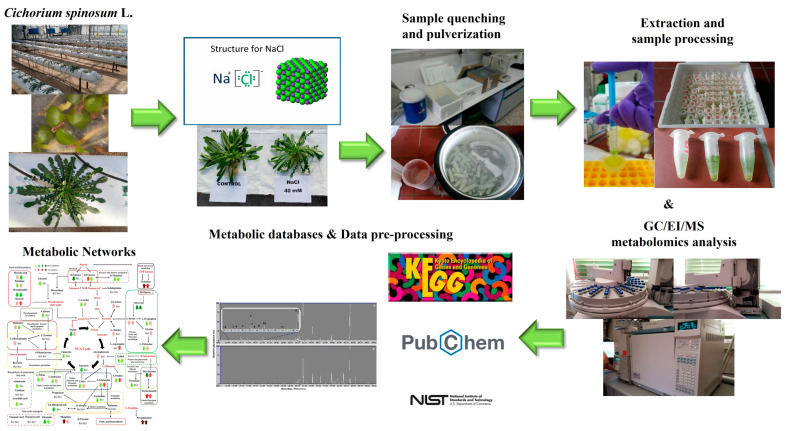
Pipeline for the dissection of the effect of two different N supply levels; low at 4 mmol L^−1^ and high at 16 mmol L^−1^, combined with three salinity levels (0.3, 20, and 40 mM) on the metabolism of *Cichorium spinosum* L. plants, employing GC/EI/MS metabolomics. An open-loop hydroponic experiment was performed using perlite as a substrate. Twelve biological replications for each *stamnagathi* ecotype were performed per treatment and the analyses were conducted at the second harvest.

**Figure 2:**
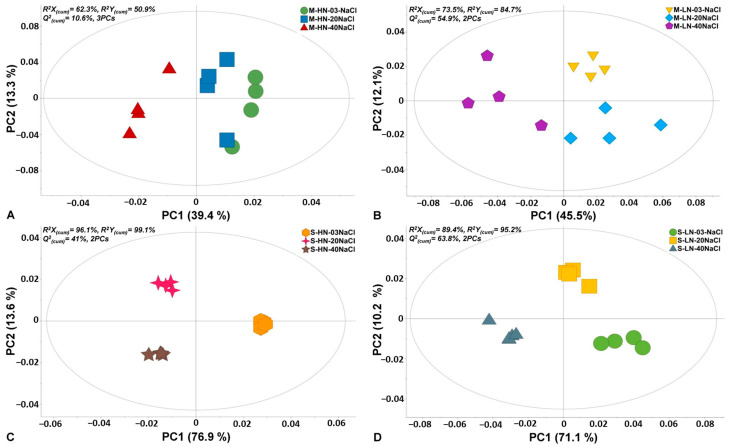
Score plots of orthogonal partial least squares-discriminant analysis (OPLS-DA) for the recorded GC/EI/MS leaf metabolomes of *Cichorium spinosum* L. (*stamnagathi*) of the montane (M) ecotype under high-N supply (HN) and non-saline treatment (0.3 mM), medium saline level (20 mM), high saline level (40 mM) (**A**), and low-N supply (LN) and the corresponding salinity treatments (**B**), and the corresponding OPLS-DA score plots of the seaside ecotype under HN (**C**) and LN (**D**) for the various salinity treatments. The ellipse represents Hotelling’s T^2^ with a 95% confidence interval. In total, twelve biological replications were performed per treatment, every three of which were pooled to provide a pooled sample. Four pooled samples and one quality control sample were analyzed per treatment. PC: principal component; Q^2^_(cum)_: cumulative fraction of the total X’s variation that can be predicted; R^2^X and R^2^Y: fraction of the sum of squares of X’s and Y’s explained variation, respectively.

The experimental and bioanalytical protocols (Figure 1) were robust, as evidenced by the high quality of the acquired chromatograms (Figure S1) and the trends observed in the obtained OPLS-DA score plots (Figure 2).

Representative data “*Cichorium spinosum L. (PMG-04-23)*” can be found in the repository of the Pesticide Metabolomics Group of the Agricultural University of Athens (https://www.aua.gr/pesticide-metabolomicsgroup/Resources/default.html). Furthermore, Heatmap and Venn diagrams were created using the online heatmapper and jvenn software (http://genoweb.toulouse.inra.fr:8091/app/index.html, accessed on 6 January 2023), respectively [52,53], to illustrate the overlap of the annotated metabolites in *stamnagathi* plants. Information was acquired from the KEGG (https://www.kegg.jp/, accessed on 6 January 2023), PubChem (https://pubchem.ncbi.nlm.nih.gov, accessed on 6 January 2023), and National Institute of Standards and Technology (NIST) (https://www.nist.gov, accessed on 6 January 2023) databases.

## 3. Results

### 3.1. Summary of the GC/EI/MS Metabolomics Analysis

Analysis of the chromatograms resulted in the discovery of 180 reproducibly detected metabolite features. Representative features that were annotated at different levels are displayed in Table S1. Annotated metabolites belong to AAs, organic acids, carboxylic acids, and fatty acids, and related information is provided. OPLS-DA plots (Figure 2) demonstrated a strong discrimination between the different treatments of the two *stamnagathi* ecotypes. In all cases, except for the seaside ecotype that was treated with high N levels (Figure 2C), the highest salinity stress of 40 mM had the greatest impact on the metabolism of the plants. Additionally, based on the observed trends, exposure of the plants to 20 mM of NaCl seems to have a greater impact on the seaside than the montane ecotype under high N levels.

**Figure 3:**
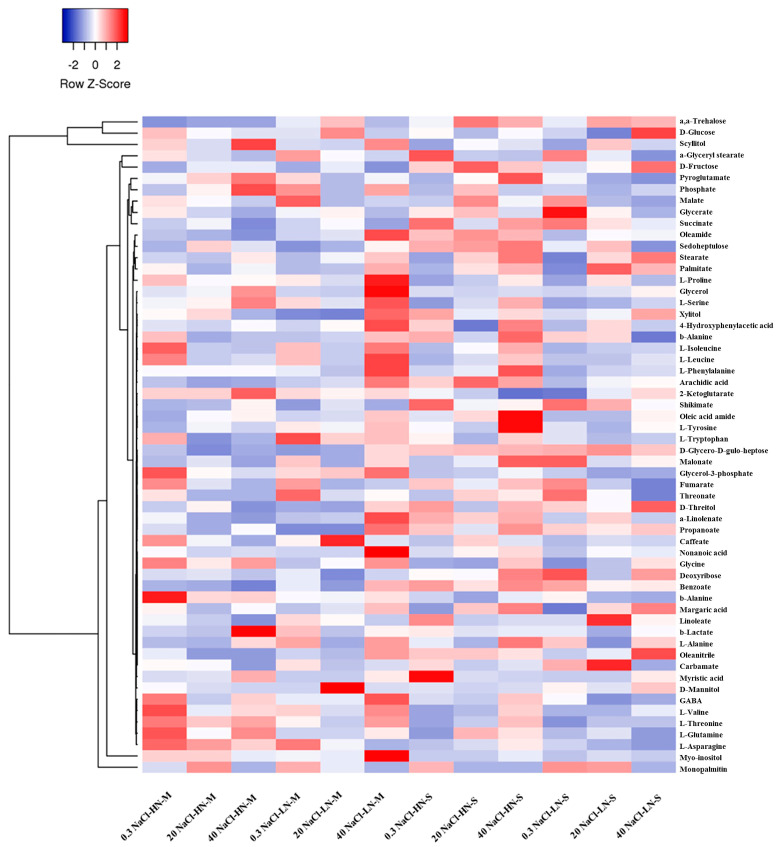
Visualization of fluctuations in the recorded GC/EI/MS leaf metabolomes of *Cichorium spinosum* L. following treatments using a heat map. In this figure, the two contrasting ecotypes (M denotes montane and S the seaside ecotype), the two different total-N levels in the supplied nutrient solution (NS) (4 mM as low N (LN) and 16 mM as high N (HN)), and three salinity (NaCl) levels, (0.3 as non-saline, 20 mM as medium saline, and 40 mM as high saline) are shown. Blue and red blocks indicate the metabolites which detected in lower and higher levels, respectively. Each heatmap block shows the average value of four replicates.

In addition, to provide a global overview of the alterations in the *stamnagathi* metabolism caused by the treatments, a cluster heat map was constructed based on the annotated metabolites (Figure 3). In total, 59 metabolites belonging to AAs, carbohydrates, carboxylic acids, and fatty acids were substantially affected by the applied treatments.

**Figure 4:**
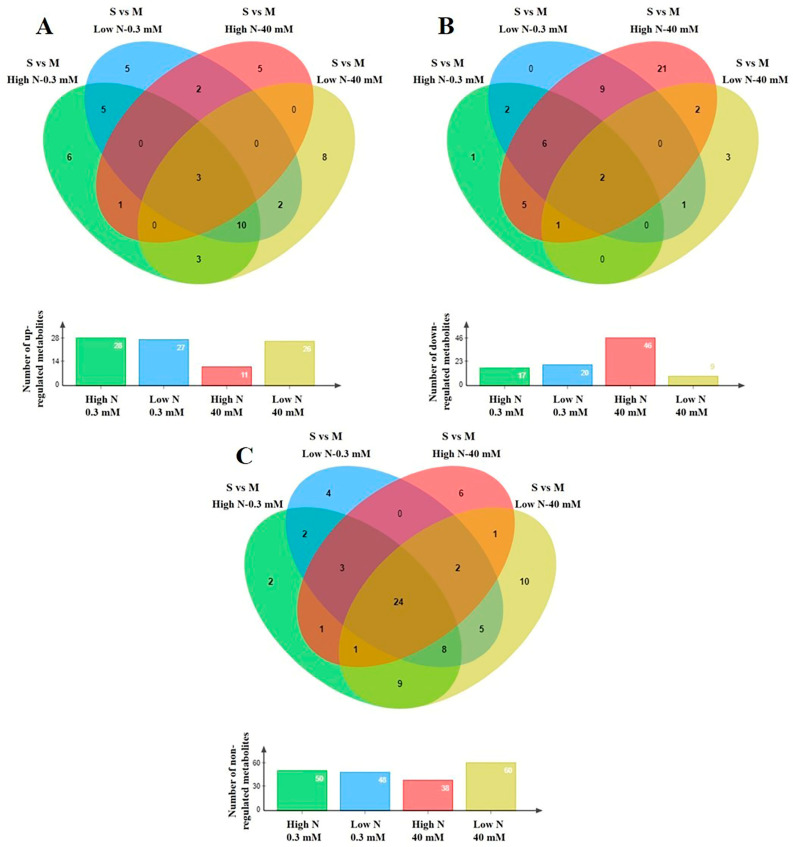
Four-way Venn diagrams of non-saline (control at 0.3 mM NaCl) and saline (the highest level at 40 mM NaCl) treatments indicating the total number of annotated primary metabolites in *stamnagathi* (*Cichorium spinosum* L.) plants which were (**A**) up-regulated, (**B**) down-regulated, and (**C**) non-regulated in the comparison between the two contrasting ecotypes (S vs. M) growing under two different N-supply levels, 4 mM denoted as low N (LN) and at 16 mM denoted as high N (HN), using GC/EI/MS.

**Figure 5:**
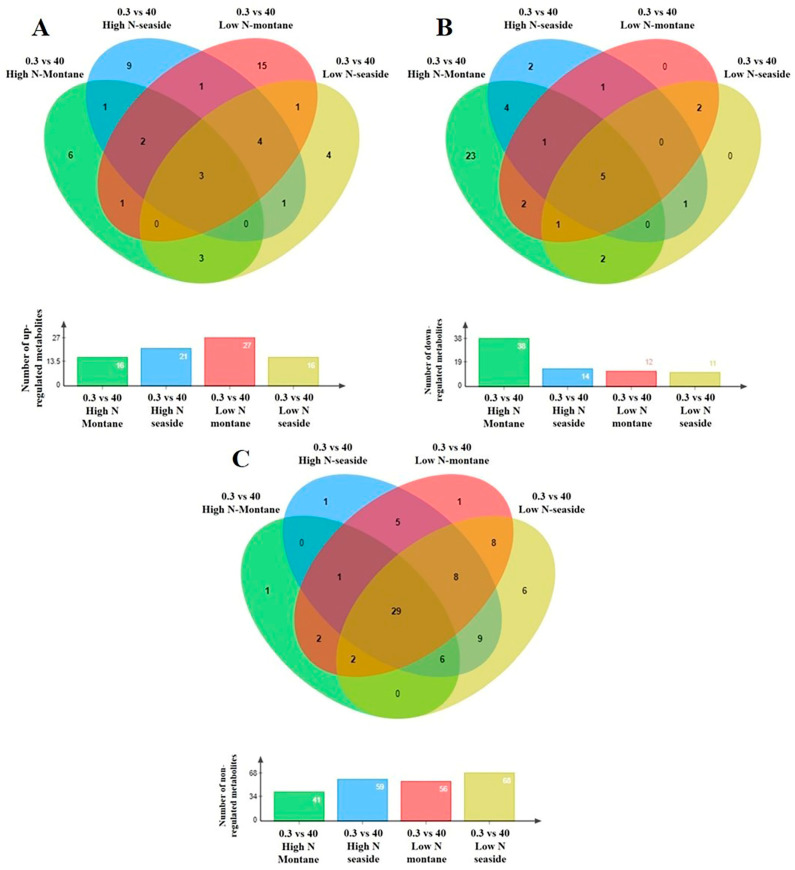
Four-way Venn diagrams of each *stamnagathi* (*Cichorium spinosum* L.) ecotype separately (the montane ecotype grown spontaneously in a mountainous area of Crete or the seaside ecotype originating from a coastal area of the same island) subjected to two different N supply levels, (4 mM, denoted as low N (LN) and 16 mM denoted as high N (HN)), indicating the total number of the annotated primary metabolites in *stamnagathi* (*Cichorium spinosum* L.) plants which were (**A**) up-regulated, (**B**) down-regulated, and (**C**) non-regulated in the comparison between the non-saline and the highest salinity treatments (0.3 vs. 40), using GC/EI/MS.

Complementary to the multivariate analysis, the constructed heatmap revealed the patterns of fluctuation of the metabolite levels among treatments. In particular, in the montane ecotype, a low N supply level combined with a higher level of salinity (40 mM) in the nutrient solution strongly differentiated the various metabolites, while in the seaside ecotype, the differentiation was higher under a high total N. It is evident that, regardless of the N source (LN or HN), the content of the identified metabolites was less affected by the low salinity levels (0.3 and 20, respectively) compared to the highest level of 40 mM. The total number of the annotated primary metabolites that were included in the analysis was 180 (Figure 4 and Figure 5). The impact of the applied treatments on *stamnagathi’s* primary metabolism was evaluated by analyzing the fluctuations of its metabolites, which were categorized into different chemical groups (Figures S2–S5). This approach aims to provide both a robust biological interpretation of the plants’ responses to the treatments and a global overview of the impact of the different stresses applied on the plants’ metabolic function.

Plants of the montane ecotype exhibited a substantial increase in the total number of primary metabolites (mainly participating in functions such as carbohydrate, AA, energy, and lipid metabolism) when grown under a high total N at high salinity conditions (Figure 4B), while responding differently under low N conditions (Figure 4A–C). The results clearly show that the metabolism of the montane ecotype, under a high total N supply at the highest salinity level of 40 mM, was generally disturbed and decreased (Figure 5B). On the other hand, the levels of a large number of the recorded primary metabolites of various metabolic functions increased or remained unaffected in the plants originating from the seaside area (Figure 5A,C).

The levels of most metabolites that belong to AAs, carbohydrates, and carboxylic acids, as well as to fatty acids were detected in lower levels (decreased) in the montane ecotype compared to those from the seaside area (Figures S2Β–S5B) when exposed to a high total N under high salinity conditions (40 mM). On the other hand, the lowest level of total N supply did not substantially alter the biosynthesis of carbohydrates or carboxylic and fatty acids, nor to a lesser extent the AA group, as most of the metabolites in these chemical groups were not affected (Figures S2–S5). An intriguing observation is that the coastal-marine (seaside) ecotype exhibited negligible changes across all investigated groups regardless of whether the plants were subjected to a high or low N supply under high salinity (40 mM). Conversely, the montane ecotype demonstrated distinct variations in the levels of AAs, carbohydrate, carboxylic acid, and fatty acids (Figures S2Β–S5Β).

**Figure 6:**
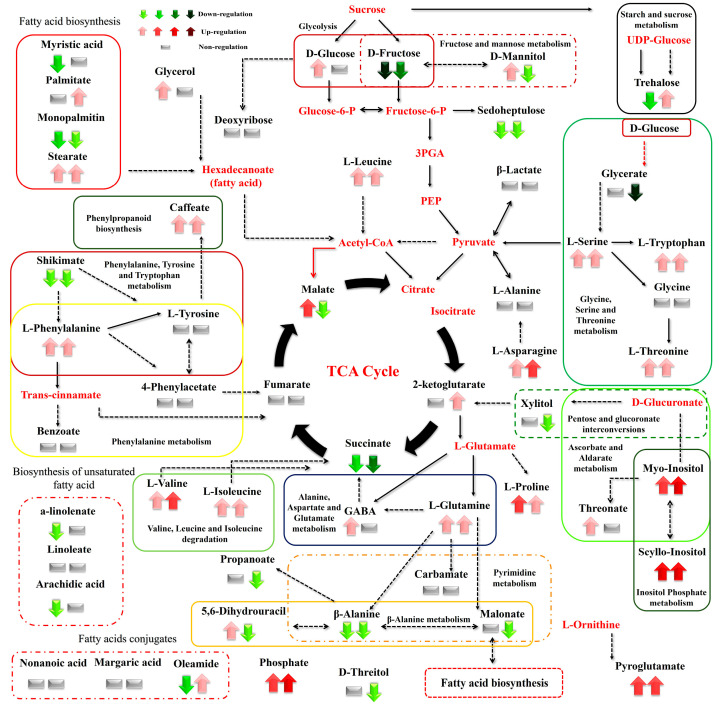
Differences between the metabolite levels of the *Cichorium spinosum* seaside (S) and montane (M) ecotypes that were grown under a high total N and non-saline conditions of 0.3 mM (left arrow or block below metabolites). In this figure, the effect of a low-N supply level in plants grown under non-saline conditions of 0.3 mM on their metabolic response (right arrow or block below metabolites) can be seen. The undetected metabolites are presented in bold red font. Red or green colors correspond to metabolites whose relative composition increased or decreased, respectively, in the montane (M) ecotype under either a high-N or low-N supply. Gray color blocks denote metabolites whose levels were not substantially altered (gray blocks below metabolites). Solid arrows are used to show the subsequent steps of a biosynthetic pathway, whereas dashed arrows show the multi-step links (TCA: tricarboxylic acid cycle; PEP: phosphoenolpyruvate; GABA: *γ*-aminobutyric acid; UDP-Glucose: uridine diphosphate glucose; 3PGA: 3-phosphoglycerate).

**Figure 7:**
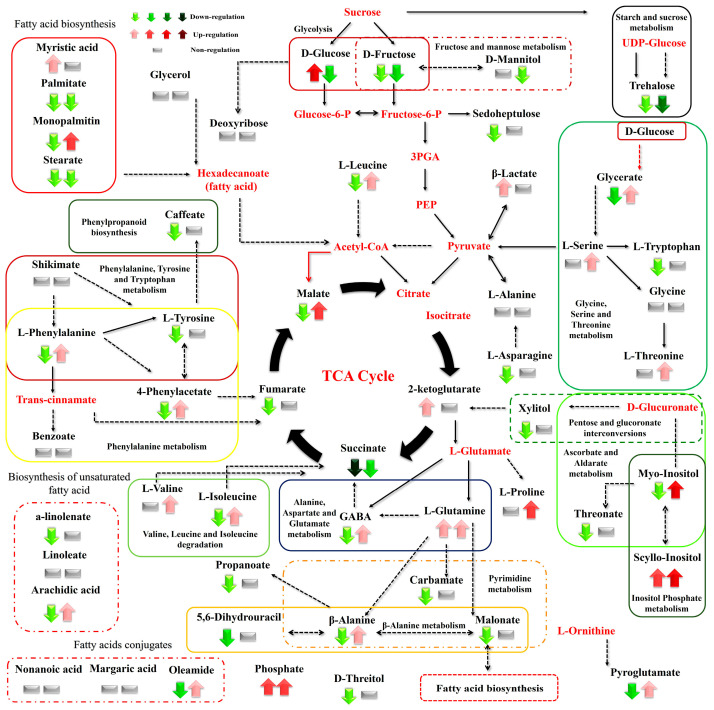
Differences between the metabolite levels of the *Cichorium spinosum* seaside (S) and montane (M) ecotypes that were grown under a high total N and high-saline conditions of 40 mM (left arrow or block below metabolites). In this figure, the effect of a low-N supply level in plants grown under high saline conditions of 40 mM on their metabolic response (right block below metabolites) can be seen. The undetected metabolites are presented in bold red font. Red or green colors correspond to metabolites whose relative composition increased or decreased, respectively, in the montane (M) ecotype at either a high-N or low-N supply under saline conditions. Gray color blocks denote metabolites whose levels were not substantially altered (gray blocks below metabolites). Solid arrows are used to show the subsequent steps of a biosynthetic pathway, whereas dashed arrows show the multi-step links (TCA: tricarboxylic acid cycle; PEP: phosphoenolpyruvate; GABA: *γ*-aminobutyric acid; UDP-Glucose: uridine diphosphate glucose; 3PGA: 3-phosphoglycerate).

**Figure 8:**
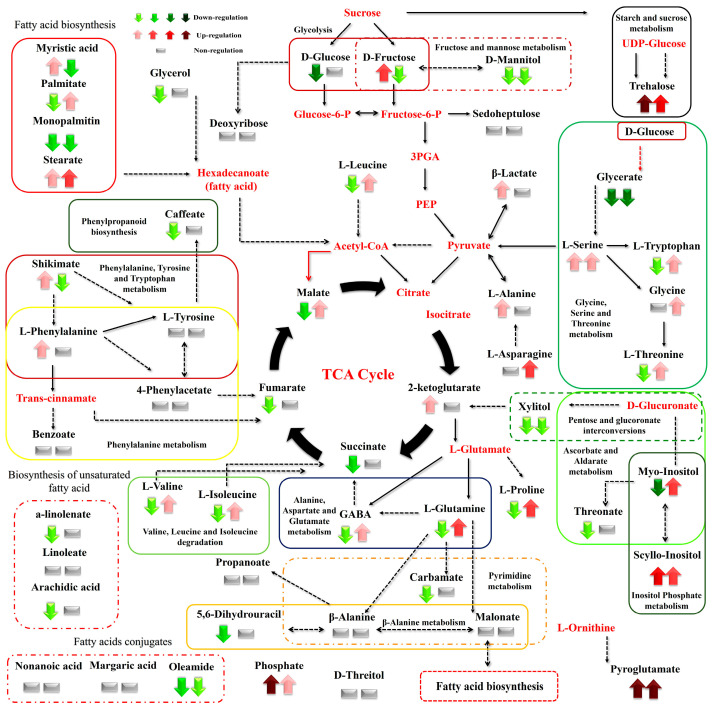
Differences between the metabolite levels of the *Cichorium spinosum* montane (M) ecotype (left arrow or block below) and seaside (S) ecotype (right arrow or right block) that were grown under a high total N supply and non-saline or high saline conditions of 0.3 and 40 mM (0.3 vs. 40), respectively. In the figure, the effect of a high total N supply combined with non-saline and high saline conditions of 0.3 and 40 mM (0.3 vs. 40), respectively, on the metabolic response of the seaside ecotype (right arrow or block below metabolites) can be seen. The undetected metabolites are presented in bold red font. Red or green colors correspond to metabolites whose relative composition was increased or decreased, respectively, in the highest saline treatment in either the montane or seaside plants. Gray color blocks denote metabolites whose levels were not substantially altered (gray blocks below metabolites). Solid arrows are used to show the subsequent steps of a biosynthetic pathway, whereas dashed arrows are used to show the multi-step links (TCA: tricarboxylic acid cycle; PEP: phosphoenolpyruvate; GABA: *γ*-aminobutyric acid; UDP-Glucose: uridine diphosphate glucose; 3PGA: 3-phosphoglycerate).

**Figure 9:**
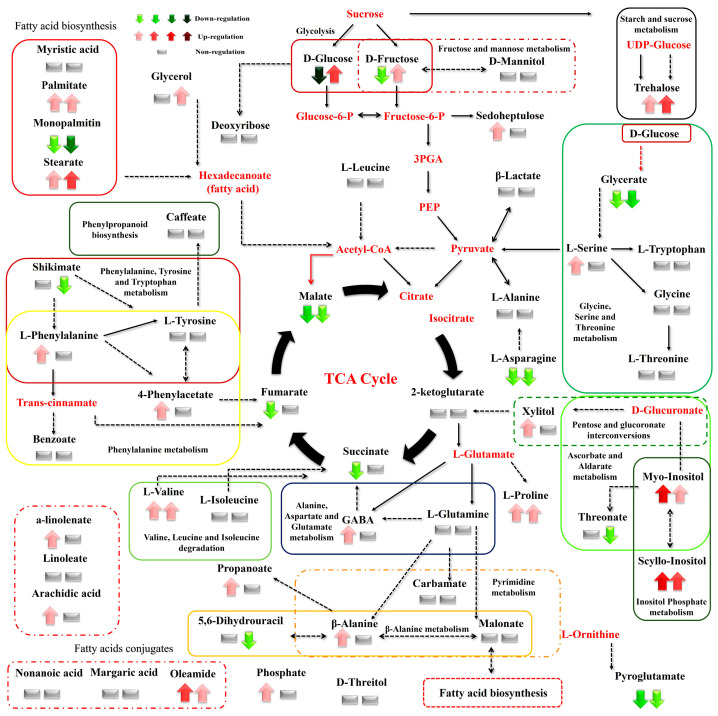
Differences between the metabolite levels of the *Cichorium spinosum* montane (M) ecotype (left arrow or left block below) and seaside (S) ecotype (right arrow or right block) that were grown under a low total N supply and non-saline or high saline conditions of 0.3 and 40 mM (0.3 vs. 40), respectively. In this figure, the effect of the low total N supply combined with non-saline and high saline conditions of 0.3 and 40 mM (0.3 vs. 40), respectively, on the metabolic response of the seaside ecotype (right arrow or block below metabolites) can be seen. The undetected metabolites are presented in bold red font. Red or green colors correspond to metabolites whose relative composition was increased or decreased, respectively, in the highest saline treatment in either the montane or seaside plants. Gray color blocks denote metabolites whose levels were not substantially altered (gray blocks below metabolites). Solid arrows are used to show the subsequent steps of a biosynthetic pathway, whereas dashed arrows are used to show the multi-step links (TCA: tricarboxylic acid cycle; PEP: phosphoenolpyruvate; GABA: *γ*-aminobutyric acid; UDP-Glucose: uridine diphosphate glucose; 3PGA: 3-phosphoglycerate).

Several metabolites involved in various biosynthetic pathways of the plant were detected. The majority of the annotated metabolites belonged to AAs, carbohydrates, and carboxylic and fatty acids (Figure 6, Figure 7, Figure 8 and Figure 9 and Figures S2–S5). De novo metabolite networking was performed to interpret the results biologically and gain insights into the fluctuations of plant metabolites in response to the various treatments. The plant treatments resulted in a general disturbance of their metabolism, as several AAs, carbohydrates, fatty acids, and carboxylic acids were detected in higher or unchanged levels in the seaside-origin plant ecotype compared to those of montane origin.

### 3.2. Effect of N Supply and Salinity Level on the Amino Acids (AA) of Stamnagathi

The biosynthesis of AAs was significantly disrupted by both the total N level and the salinity conditions tested. More specifically, high salinity decreased the levels of Pro—an important osmolyte for stress tolerance—in the montane ecotype, while the reverse was the case for the seaside ecotype (Figure 8). Additionally, Pro showed a slight increase under a low total-N supply and high salinity in both examined ecotypes (Figure 9). However, except for the saline treatment under a 16 mM N supply, Pro tended to exhibit a significant increase in both comparisons between the two contrasting ecotypes (Figure 6 and Figure 7). In contrast to Pro, the levels of phenylalanine (Phe), tyrosine (Tyr), leucine (Leu), tryptophan (Trp), valine (Val), isoleucine (Ile), threonine (Thr), glutamine (Gln), glycine (Gly), and alanine (Ala) were either not substantially affected by the combined stress conditions or were affected to a lesser extent (Figure 8). Interestingly, most of the detected AAs also showed a significant response under stress and non-stress conditions, as presented in the metabolite networks established (Figure 6, Figure 7 and Figure 8). The non-protein amino acid GABA increased in plants originating from a seaside habitat and grown under saline stress conditions and a high total N supply, while the reverse was the case for the montane plants (Figure 8). However, under the combined stress conditions, the levels of GABA were slightly increased in the montane ecotype, while they were not substantially affected in the seaside ecotype (Figure 9). An increase in the salt level of the supplied nutrient solution (NS) either up-regulated or down-regulated the biosynthesis of GABA in the montane ecotype compared to those from the seaside area, when plants were treated with either a low and high N supply, respectively (Figure 7).

### 3.3. Effect of N Supply and Salinity Level on the Carbohydrate Content

Carbohydrates such as Glu and Fru were detected in lower levels in the montane ecotype compared to the seaside area, when plants were treated with a low N and high salinity (40 mM). Moreover, Glu was higher in montane *stamnagathi* leaves with a high N supply, while Fru levels were higher in the seaside ecotype (Figure 7). The increase in salt levels from 0.3 mM to 40 mM at a low N supply resulted in decreased levels of both Glu and Fru in the montane ecotype (Figure 9). The levels of the monosaccharide sedoheptulose and the sugar alcohols D-mannitol and D-threitol were either not substantially affected by the stress and non-stress conditions or were affected to a lesser extent (Figure 6, Figure 7, Figure 8 and Figure 9). Additionally, *α,α*-trehalose was significantly lower in plants receiving a high or low N supply combined with higher salinity levels in the montane ecotype compared to plants originating from the seaside area (Figure 7). With respect to high saline levels, *α,α*-trehalose was higher in montane ecotype when plants were grown under a high N supply level and higher in the seaside ecotype when plants were subjected to a low N (Figure 8 and Figure 9). A similar trend was observed for the monosaccharide myo-inositol, which significantly increased in the seaside ecotype under a high N supply and saline conditions, while decreasing in the montane ecotype under the same conditions (Figure 8). Conversely, a decrease in the N supply from 16 mM to 4 mM under saline conditions resulted in significantly increased levels of the monosaccharide (Figure 9).

### 3.4. Effect of N Supply and Salinity Level on Carboxylic Acids, Fatty Acids, and Selected Stamnagathi Metabolites

Carboxylic acids, the intermediates of the Krebs cycle, were substantially affected by the combined N supply and saline conditions. The highest effect of both high and low total N supply under saline conditions was observed on malate, fumarate, and succinate in the montane ecotype. Moreover, increasing salt levels in the supplied NS resulted in decreased levels of both metabolites in the montane ecotype, whereas in the seaside ecotype, they were not substantially affected (Figure 8). On the other hand, 2-ketoglutarate either increased or was not affected by the examined treatments (Figure 8 and Figure 9). Additionally, a similar trend was observed for the comparison between the two contrasting ecotypes under a high N supply (Figure 7). Other carboxylic acids, such as shikimate, threonate, carbamate, and benzoate were either not affected or affected to a minor degree by the treatments applied (Figure 6, Figure 7, Figure 8 and Figure 9). Fatty acids such as a-linolenate (ALA) and linoleate (LA) were not significantly influenced by stress conditions in the seaside ecotype by the treatments applied. Conversely, in the montane ecotype, a slight increase was found under a low N supply and high salinity level, whereas the reverse was the case under a high N and saline conditions (Figure 6, Figure 7, Figure 8 and Figure 9). On the other hand, the biosynthesis of palmitate and stearate was down-regulated in the examined comparison between the montane ecotype and the seaside ecotype when the total N was either high or low in the supplied NS (Figure 7). Additionally, both ecotypes in the comparison between the different saline levels showed an increase in the concentration of these fatty acids with an increase in salt levels (Figure 9). Caffeate, from the group of phenylpropanoids, was either not influenced or influenced to a minor degree by the ecotype, N-supply level, or saline conditions. Finally, phosphate from the phosphoric acid derivatives group showed significantly higher levels in all of the examined comparisons except for the 4 mM N supply combined with 40 mM salinity (Figure 6, Figure 7, Figure 8 and Figure 9).

## 4. Discussion

Here, we applied GC/EI/MS metabolomics to elucidate the effect of N supply and salinity level on the metabolite composition of *Cichorium spinosum* L. leaves. Our multivariate analyses showed significant effects of the genetic background (Figure 2) and treatments on *stamnagathi* metabolite composition, with many annotated metabolites exhibiting substantial fluctuation.

Salt stress is primarily responsible for inducing osmotic phenomena or ion toxicity in plants, thereby leading to the inhibition of plant growth and development, oxidative stress, and nutrient deficiency [54]. It is also responsible for a wide range of alterations in plant’s physiological processions and metabolic regulations. Compatible solutes or osmolytes are a category of compounds that contain N in their structure, i.e., AAs, amines and betaines, organic acids, sugars, and polyols [55]. When plants are subjected to salinity stress, the overexpression of osmolytes plays a crucial role in protecting against osmotic phenomena by stabilizing lipid membranes, protecting essential enzymes and proteins from denaturation, shielding plants from the harmful effects of ROS [18,19], and preventing imbalances caused by osmotic stress [2,3,4]. Moreover, GABA has been shown to play a role in nitrogen remobilization and nitrogen use efficiency in plants and is involved in the regulation of N uptake and assimilation, while under low N conditions it accumulates in plant tissues [56,57]. The results of our study indicate that osmolytes, including Pro, GABA, soluble sugars, carboxylic acids, etc., exhibit differential responses under saline and high N supply conditions. In particular, a large number of those osmoprotectants were either up-regulated or not substantially altered in the seaside ecotype, indicating a higher tolerance to salt tress compared to the montane ecotype. This indicates that the seaside ecotype has evolved mechanisms to cope with the adverse effects of salt stress and high N levels, which may have resulted from its adaptation to the coastal environment where salt stress and nutrient imbalances occur. Previous studies have shown that Pro plays a crucial role in intercellular osmotic adjustment [58] and can act as a signaling molecule in response to stress [59]. Pro’s roles also include the stabilization of cell membranes and proteins [60] and the maintenance of plant turgor [61,62]. In this study, Pro and GABA levels increased in the seaside ecotype when subjected to salinity stress and high N, while the reverse was the case for the montane ecotype. Similar results were found for Leu, Ile, Trp, Gul, Val, and Thr. The increasing content of Pro in *stamnagathi* plants exposed to high salinity levels is consistent with previous findings in *Hordeum vulgare* [63,64]. Furthermore, it has been suggested that Pro may be involved in K^+^ homeostasis by protecting against the leakage of K^+^ in NaCl-treated cells [65]. The relationship between Pro levels and N levels in plants is not straightforward and can depend on various factors, including the plant species and the type of N source [66,67]. In some studies, high N levels have been associated with increased Pro levels, while in others, no significant relationship was observed. Overall, the increase in Pro levels under high salinity and high N may be part of the plant’s adaptive response to cope with these stress factors. The accumulation of Pro has also been reported in the study of Behr et al. [68] under a double stress of salinity and hypoxia, suggesting that Pro may act either as a ROS scavenger or play a role in pH adjustment and the redox state of the stressed cell. According to Zhang et al. [69] differences were found in the AAs among wild and cultivated soybean genotypes grown under control and salinity stress. More specifically, the amino acids Ser, Ile, Ala, Gly, Phe, Val, Thr, and Tyr were higher in the leaves of the cultivated genotype compared to the wild type. A recent study of Hildebrandt et al. [70] reported that the oxidation of AAs such as Val, Leu, and Ile can directly enhance electrons into the mitochondrial electron transport chain, clearly indicating their role in mitochondrial metabolism and ATP production. In our study, under salt-stress conditions and a high total-N supply, these AAs were decreased in the montane ecotype and increased in the seaside ecotype. Besides their role in energy production, AAs also act as signaling molecules or precursors for the synthesis of other secondary metabolites and phytohormones during stress conditions. Tyr and Phe, which are derived from the shikimate pathway, act as precursors for alkaloids and other secondary metabolites or as ROS scavengers [71,72]. In *S. persica* plants, the increasing trend of these AAs under salinity may be correlated with an increase in shikimate pathway activity, thereby leading to the biosynthesis of Phe [73].

Salinity stress causes crucial disorders in plants, including lower photosynthetic rates, inhibition of nitrogen assimilation, and disrupted cell division, leading to problematic growth and development [74]. An exogenous application of salt can also restrict leaf growth, disrupt ionic homeostasis and reduce stomatal conductance [75], promote dry weight and proline accumulation, prevent cell dehydration, and activate crucial antioxidant enzymes [76]. In order to ameliorate impact of stress conditions, sugars can act as osmolytes [77]. Essential sugars such as Suc, Fru, and Glu—when overexpressed in plants under saline conditions—provide protection against osmotic phenomena, are involved in carbon storage and osmotic homeostasis regulation, and serve as ROS scavengers [78]. In the present study, Glu decreased only in the montane ecotype grown under the combined stress (low N supply and high salinity), clearly indicating its susceptibility to stress. Silva-Ortega et al. [35] referred to the theory that when plants are exposed to stress conditions, including salinity, crucial compatible solutions such as sugars could be enhanced. Similar trends were observed at a high N level in the supplied NS, where Glu also declined only for the montane ecotype. Moreover, α-α-trehalose and Fru were higher in the seaside ecotype compared to the montane ecotype. According to Hu et al. [76], a lower application of Glu in wheat seedlings boosts seed germination under stress conditions, while higher concentrations inhibit it [79]. In salt-sensitive plants such as rice, Glu and Fru serve as osmoprotectant compounds and scavengers of ROS [80]. Their role is associated with ROS anabolism and catabolism, such as the ROS scavenger-involved pathway of oxidative pentose phosphate [81]. Finally, in wheat seedlings, an exogenous application of Glu under salt-stress conditions results in a decline in Na^+^ concentrations, a higher accumulation of K^+^, and the maintenance of ionic homeostasis at a normal level [82]. However, it should be noted that in some cases, the response of specific carbohydrates to salinity stress may be species-dependent [83]—as in the study of Hafiz Che-Othman et al. [84], in which salinity stress in wheat leaves increased all sugars except for Glu.

There is a strong hypothesis that suggests that plants can maintain cellular osmolarity, energy metabolism, and ROS scavenging by accumulating sugars and sugar alcohols [72,85]. However, under saline stress conditions, an accumulation of sugars is also utilized by plants as an immediate energy source to achieve restoration in their growth by stabilizing macromolecules [86]. These compounds are also involved in the sugar-sensing system, a system which regulates the expressions of crucial genes responsible for photosynthesis and respiration, providing plants with a better response against abiotic stresses [87]. The critical role of myo-inositol (sugar alcohol) has already been referred to in previous studies. It is a primary metabolite that can provide salt tolerance by protecting the cells against harmful ROS, regulating osmotic balance, and there is evidence that it has a role in the storage and transport of auxins [88]. In the present study, under a high total-N supply and saline treatment, myo-inositol enhanced its levels only in the case of seaside ecotype (up-regulation), while the reverse was the case for the montane (down-regulation). This indicates that the seaside ecotype is better adapted to cope with a high total-N supply and salinity stress compared to the montane ecotype. Moreover, the accumulation of myo-inositol in the seaside ecotype suggests its importance in protecting cells against ROS and regulating osmotic balance under stress conditions. These findings are consistent with previous studies that have shown the beneficial effects of myo-inositol on plant growth and stress tolerance. For instance, in quinoa, exogenous application of myo-inositol has been found to enhance salt tolerance by regulating the expression of stress-responsive genes and increasing the activities of antioxidant enzymes [89]. Similarly, and in agreement with the present study, Gong et al. [39] showed greater levels of myo-inositol in the halophyte *Thellungiella halophila* under saline conditions, as well as in the halophyte *Chenopodium quinoa*. On the other hand, at a low N supply, myo-inositol was observed to increase in both of the examined ecotypes. This suggests that myo-inositol accumulation in response to salinity stress may depend on the interaction between nutrient availability and salt stress. In the case of a high total-N supply, the seaside ecotype may have been able to increase its myo-inositol levels as a strategy to cope with salt stress, while the montane ecotype may have down-regulated its myo-inositol levels due to limitations on energy and resource allocation. At a low N supply, both ecotypes may have increased myo-inositol levels as a response to the combined stress of nutrient deficiency and salinity. Furthermore, the increase in myo-inositol content under a low N supply in both ecotypes may suggest that myo-inositol plays a role in nitrogen use efficiency and N stress responses in plants. These findings highlight the complex and dynamic nature of plant responses to abiotic stress, which can be influenced by multiple environmental factors.

Halophytes are plants that can thrive under high EC (exceeding 150 mM) due to their adaptive mechanisms of salt stress tolerance. These mechanisms are characterized by a reduced waste of energy during salt-stress and the protection of physiological processes such as photosynthesis. Other mechanisms are associated with the activation of stress-related enzymes which provide protection against harmful ROS (H_2_O_2_, O_2_, and OH^−^) through increased antioxidant activity. In this study, the levels of the carboxylic acids (fumarate, succinate, and a-ketoglutarate) in the seaside ecotype were not substantially affected by stress conditions when plants were exposed to high saline conditions (40 mM). In contrast, in the montane ecotype, carboxylic acids either increased or mainly decreased, indicating that the seaside plants are capable of conserving energy and probably protecting their physiological processes, giving them a tolerant-response under stress conditions. According to Borrelli et al. [6], metabolites from the TCA cycle in different examined wheat varieties showed significant differences depending on the genotype. Increased salinity levels resulted in decreased carboxylic acid content regardless of the genotype when plants were exposed to 50 mM NaCl. On the other hand, at higher concentrations of NaCl, the only genotype that managed to maintain the TCA cycle unaffected was “Cappelli”. For that reason, these metabolites (intermediates of the Krebs cycle) can be exported and used as carbon skeletons for the synthesis of salt-induced substances. Due to this process, plants do not possess enough energy in the form of ATP and reduction equivalents such as NADH and FADH_2_, especially in the case of the highest salinity level. This unavailable energy can easily lead to critical impacts on plant growth and development in the examined genotypes which are sensitive. In this study, a decrease in the metabolite content from the TCA cycle was observed for the montane ecotype at the highest salt treatment, with the exception of a-ketoglutarate. On the other hand, the seaside ecotype showed a better maintenance of the TCA cycle, indicating a higher tolerance of this ecotype to salinity. A decrease in the metabolites of the TCA cycle (malate, succinate, 2-ketoglutarate) with an increase in salt levels [90] is well documented. As has already been mentioned, the reduction in the intermediates of the TCA cycle provides less available energy in the form of NADH, FADH_2_, and ATP, which negatively affects plant growth. Indeed, salt-resistant maize hybrids are more capable of growing with less energy compared to salt-sensitive hybrids [90]. In our case, the response of the seaside ecotype seems to be similar to that of a salt-tolerant plant due to its ability to maintain energy. According to Wu et al. [64], who also examined the impact of salinity stress on different barley varieties (wild and cultivated), metabolites from the TCA cycle (citrate, 2-ketoglutarate, fumarate, malate, and succinate) were reduced under salinity conditions. According to Kiani-Pouya et al. [72], the reduction in organic acids, especially intermediates of the TCA cycle, under salinity treatments leads to a decrease in TCA cycle activity. This reduction causes an increase in the demand for carbon structures to produce other compounds required to adjust the osmotic phenomena that derive from salt stress. Similar to our results, high saline conditions can lead to lower concentrations of the detected carboxylic acids in other plants, i.e., *Thellungiella halophila*, *Suaeda salsa*, and *Chenopodium quinoa* [39,72,91].

The cell membrane in plants plays a critical role in regulating the transport of ions and larger molecules. Since plants are exposed to abiotic stresses such as salinity, they have developed (and/or adopted) protective mechanisms such as the production of antioxidant enzymes, i.e., superoxide dismutase and ascorbate peroxidase to protect themselves from impending oxidative stress and defend against ROS. The plasma membrane is the first part of the cell that salt reaches, and the role of the membrane lipids and transport proteins in maintaining the permeability of this membrane is critical in responding to salinity stress. This correspondence constitutes one of the primary responses against this stress [92]. In many cases, the impact of salt stress conditions on plasma membrane lipids, i.e., sterols and fatty acids might be the reason behind alterations in membrane fluidity and permeability, and therefore a possible mechanism against stress [93,94]. According to Liu et al. [95], the composition of lipids and unsaturated fatty acids can affect membrane fluidity and structure. Furthermore, plant’s tolerance to salinity stress has been observed to be associated with increased levels of unsaturated fatty acids. In addition, an increase in the levels of unsaturated fatty acids in membrane lipids has been shown to reduce the inhibition on PSII that is enhanced by salt stress treatments in the halophyte *Thellungiella halophile* [39]. Similar results have been obtained for tobacco plants, where an increase in 18:3 fatty acids levels has been associated with a greater tolerance to stress [96]. A typical example of the importance of fatty acid metabolism in plant stress responses is the process of β-oxidation. This process, which is the primary means of fatty acid decomposition, occurs in mitochondria and provides the plant with a significant amount of energy that is available for various metabolic activities. In addition to providing energy, β-oxidation plays a crucial role in plant survival under abiotic stress conditions by breaking down fatty acids molecules to generate acetyl-CoA, which can enter the TCA cycle to produce energy. This amount of energy is capable of fueling various metabolic activities [97,98]. In our study, stearate, palmitate, and monopalmitin were down-regulated in the montane ecotype compared to the seaside ecotype, clearly indicating the susceptibility of the former to salinity stress. The findings of Zhang et al. [69] suggest that the reduction in stearate and palmitate under salt stress in soybean seedlings may indicate that β-oxidation is not the main metabolic pathway. Similarly, in *S. persica* plants, the level of stearate decreased while palmitate increased in response to salt stress [73], indicating that *S. persica* reprogrammed the fatty acid composition to confer salinity stress tolerance.

## 5. Conclusions

Here, we observed substantial differences in the biosynthesis of crucial osmolytes such as sugars, Pro, and organic acids of the TCA cycle between two contrasting *stamnagathi* ecotypes. These differences suggest that the seaside area ecotype may had developed different mechanisms of tolerance under salt stress compared to the corresponding montane ecotype plants. More specifically, under high saline stress and high N supply conditions, GABA, Pro, malate, succinate, fumarate, etc., exhibited differential fluctuation, indicating that the seaside ecotype has developed adaptation mechanisms regulated by primary metabolites. These findings highlight the potential for utilizing this ecotype as a “control plant” in integrated breeding programs. The metabolic approach which utilizes GC/EI/MS can (i) illustrate the response of non-cultivated plants under high saline or combined treatments, (ii) enable the identification of possible stress tolerance mechanisms and the role of the detected metabolites, not only in the leaves but also in the root system, for further investigation, and (iii) constitute a crucial step in investigating gene-related, hormone-related, and enzyme-related compounds associated with salt stress originating from local or wild landraces.

## Data Availability

A representative data set can be accessed via the website of the Pesticide Metabolomics Group at https://www.aua.gr/pesticide-metabolomicsgroup/Resources/default.html [*Chichorium spinosum* (PMG-04-23)].

## Supplementary Materials

The following supporting information can be downloaded at: https://www.mdpi.com/article/10.3390/biom13040607/s1, Figure S1: Representative GC/EI/MS total ion chromatograms (TIC) of the endo-metabolomes of *Cichorium spinosum* L. (*stamnagathi*) leaves of the montane (M) and seaside (S) ecotypes, under high (HN) and low (LN) N supply and medium saline level (20 mM NaCl). In the upper TIC, magnification of the red dashed area and annotations for representative metabolites are displayed; Figure S2: Four-way Venn diagrams of non-saline (control at 0.3 mM NaCl) and saline (highest level at 40 mM NaCl) treatments indicating the total number of the annotated amino acids in *stamnagathi* (*Cichorium spinosum* L.) plants which were (A) up-regulated, down-regulated, and non-regulated in the comparison between the two contrasting ecotypes (S vs. M) growing under two different N supply levels, a low level at 4 mM as low N and the highest at 16 mM as high N, using GC/EI/MS, as well as (B) in the comparison of non-saline and saline treatments (0.3 vs. 40) for each ecotype separately. Each Venn diagram and its histogram was performed by the Jvenn online website (http://genoweb.toulouse.inra.fr:8091/app/index.html, accessed on 6 January 2023) [53]; Figure S3: Four-way Venn diagrams of non-saline (control at 0.3 mM NaCl) and saline (highest level at 40 mM NaCl) treatments indicating the total number of the annotated carbohydrates in *stamnagathi* (*Cichorium spinosum* L.) plants which were (A) up-regulated, down-regulated, and non-regulated in the comparison between the two contrasting ecotypes (S vs. M) growing under two different N supply levels, a low level at 4 mM as low N and the highest at 16 mM as high N, using GC/EI/MS, as well as (B) in the comparison of non-saline and saline treatments (0.3 vs. 40) for each ecotype separately. Each Venn diagram and its histogram was performed by the Jvenn online website (http://genoweb.toulouse.inra.fr:8091/app/index.html, accessed on 6 January 2023) [53]; Figure S4: Four-way Venn diagrams of non-saline (control at 0.3 mM NaCl) and saline (the highest level at 40 mM NaCl) treatments indicating the total number of the annotated carboxylic acids in *stamnagathi* (*Cichorium spinosum* L.) plants which were (A) up-regulated, down-regulated, and non-regulated in the comparison between the two contrasting ecotypes (S vs. M) growing under two different N supply levels, a low level at 4 mM as low N and the highest at 16 mM as high N, using GC/EI/MS, as well as (B) in the comparison of non-saline and saline treatments (0.3 vs. 40) for each ecotype separately. Each Venn diagram and its histogram was performed by the Jvenn online website (http://genoweb.toulouse.inra.fr:8091/app/index.html, accessed on 6 January 2023) [53]; Figure S5: Four-way Venn diagrams of non-saline (control at 0.3 mM NaCl) and saline (highest level at 40 mM NaCl) treatments indicating the total number of the annotated fatty acids in *stamnagathi* (*Cichorium spinosum* L.) plants which were (A) up-regulated, down-regulated, and non-regulated in the comparison between the two contrasting ecotypes (S vs. M) growing under two different N supply levels, a low level at 4 mM as low N and the highest at 16 mM as high N, using GC/EI/MS, as well as (B) in the comparison of non-saline and saline treatments (0.3 vs. 40) for each ecotype separately. Each Venn diagram and its histogram was performed by the Jvenn online website (http://genoweb.toulouse.inra.fr:8091/app/index.html, accessed on 6 January 2023) [53]; Table S1: Metabolite features of *Cichorium spinosum* L. (*stamnagathi*) GC/EI/MS total ion chromatograms (TIC) annotated at various identification levels.

## References

[1] Akhtar S., Sangam S., Chattopadhyay T., Naik A., Solankey S.S., Solankey S.S., Kumari M. (2023). Emerging Obstacles of Vegetable Production Due to Climate Change and Mitigation Strategies. Advances in Research on Vegetable Production under a Changing Climate.

[2] Ashraf M., Foolad M.R. (2007). Roles of glycine betaine and proline in improving plant abiotic stress resistance. Environ. Exp. Biol..

[3] Hosseinifard M., Stefaniak S., Ghorbani Javid M., Soltani E., Wojtyla Ł., Garnczarska M. (2022). Contribution of Exogenous Proline to Abiotic Stresses Tolerance in Plants: A Review. Int. J. Mol. Sci..

[4] Wang X., Dai W.W., Liu C., Zhang G.X., Song W.H., Li C., Yangchen Y.C., Gao R.F., Chen Y.Y., Yan H. (2022). Evaluation of Physiological Coping Strategies and Quality Substances in Purple Sweet Potato under Different Salinity Levels. Genes.

[5] Parihar P., Singh S., Singh R., Singh V.P., Prasad S.M. (2015). Effect of salinity stress on plants and its tolerance strategies: A review. Environ. Sci. Pollut. Res..

[6] Borrelli M.G., Fragasso M., Nigro F., Platani C., Papa R., Beleggia R., Trono D. (2018). Analysis of metabolic and mineral changes in response to salt stress in durum wheat (*Triticum turgidum* ssp. durum) genotypes, which differ in salinity tolerance. Plant Physiol. Biochem..

[7] Chaves M.M., Flexas J., Pinheiro C. (2009). Photosynthesis under drought and salt stress: Regulation mechanisms from whole plant to cell. Ann. Bot..

[8] Ma Y., Dias M.C., Freitas H. (2020). Drought and Salinity Stress Responses and Microbe-Induced Tolerance in Plants. Front. Plant Sci..

[9] Munns R., Jwmes R.A., Lauchli A. (2006). Approaches to increasing the salt tolerance of wheat and other cereals. J. Exp. Bot..

[10] Seleiman M.F., Aslam M.T., Alhammad B.A., Hassan M.U., Maqbool R., Chattha M.U., Khan I., Gitari H.I., Uslu O.S., Roy R. (2022). Salinity stress in wheat: Effects, mechanisms and management strategies. Phyton.

[11] Hu Y., Schmidhalter U. (2005). Drought and salinity: A comparison of their effects on the mineral nutrition of plants. J. Plant Nutr. Soil Sci..

[12] Shahid M.A., Sarkhosh A., Khan N., Balal R.M., Shahid A., Rossi L., Gómez C., Mattson N., Nasim W., Garcia-Sanchez F. (2020). Insights into the Physiological and Biochemical Impacts of Salt Stress on Plant Growth and Development. Agronomy.

[13] Zhu Z., Wei G., Li J., Qian Q., Yu J. (2004). Silicon alleviates salt stress and increases antioxidant enzymes activity in leaves of salt-stressed cucumber (*Cucumis sativus* L.). Plant Sci..

[14] Ashraf M., Harris P.J. (2004). Potential biochemical indicators of salinity tolerance in plants. Plant Sci..

[15] Sarwar M., Anjum S., Alam M.W., Ali Q., Ayyub C.M., Haider M.S., Ashraf M.I., Mahboob W. (2022). Triacontanol regulates morphological traits and enzymatic activities of salinity affected hot pepper plants. Sci. Rep..

[16] Zhu J.K. (2002). Salt and drought stress signal transduction in plants. Annu. Rev. Plant Physiol. Plant Mol. Biol..

[17] Wang Y., Cao Y., Liang X., Zhuang J., Wang X., Qin F., Jiang C. (2022). A dirigent family protein confers variation of Casparian strip thickness and salt tolerance in maize. Nat. Commun..

[18] Hasegawa P.M., Bressan R.A., Zhu J.K., Bohnert H.J. (2000). Plant cellular and molecular responses to high salinity. Annu. Rev. Plant Physiol. Plant Mol. Biol..

[19] Nguyen H.T.T., DasBhowmik S., Long H., Cheng Y., Mundree S., Hoang L.T.M. (2021). Rapid Accumulation of Proline Enhances Salinity Tolerance in Australian Wild Rice Oryza australiensis Domin. Plants.

[20] Munns R. (2002). Comparative physiology of salt and water stress. Plant Cell Environ..

[21] Alcázar R., Bueno M., Tiburcio A.F. (2020). Polyamines: Small Amines with Large Effects on Plant Abiotic Stress Tolerance. Cells.

[22] Ghosh U.K., Islam M.N., Siddiqui M.N., Cao X., Khan M.A.R. (2022). Proline, a multifaceted signalling molecule in plant responses to abiotic stress: Understanding the physiological mechanisms. Plant Biol..

[23] Munns R., Tester M. (2008). Mechanisms of salinity tolerance. Annu. Rev. Plant Biol..

[24] Kiani R., Arzani A., Mirmohammady Maibody S.A.M. (2021). Polyphenols, Flavonoids, and Antioxidant Activity Involved in Salt Tolerance in Wheat, *Aegilops cylindrica* and Their Amphidiploids. Front. Plant Sci..

[25] Hassani A., Azapagic A., Shokri N. (2021). Global predictions of primary soil salinization under changing climate in the 21st century. Nat. Commun..

[26] Chinnusamy V., Zhu J., Zhu J.K. (2006). Salt stress signaling and mechanisms of plant salt tolerance. Genet. Eng..

[27] Benjamin J.J., Lucini L., Jothiramshekar S., Parida A. (2019). Metabolomic insights into the mechanisms underlying tolerance to salinity in different halophytes. Plant Physiol. Biochem..

[28] Hasegawa P.M. (2013). Sodium (Na^+^) homeostasis and salt tolerance of plants. Environ. Exp. Bot..

[29] Shabala S., Pottosin I. (2014). Regulation of potassium transport in plants under hostile conditions: Implications for abiotic and biotic stress tolerance. Physiol. Plantar..

[30] Munns R., James R.A., Gilliham M., Flowers T.J., Colmer T.D. (2016). Tissue tolerance: An essential but elusive trait for salt-tolerant crops. Funct. Plant Biol..

[31] Rhodes D., Hanson A.D. (1993). Quaternary ammonium and tertiary sulphonium compounds in higher plants. Annu. Rev. Plant Physiol. Plant Mol. Biol..

[32] Kishor P.B.K., Hong Z., Miao G.H., Hu C.A.A., Verma D.P.S. (1995). Over-expression of [delta]-pyrroline-5-carboxylate synthetase increases proline production and confers osmotolerance in transgenic plants. Plant Physiol..

[33] Verbruggen N., Hermans C. (2008). Proline accumulation in plants: A review. Amino Acids.

[34] Amini F., Ehsanpour A. (2005). Soluble proteins, proline, carbohydrates and Na^+^/K^+^ changes in two tomato (*Lycopersicon esculentum* Mill.) cultivars under in vitro salt stress. Am. J. Biochem. Biotechnol..

[35] Silva-Ortega C.O., Ochoa-Alfaro A.E., Reyes-Agüero J.A., Aguado-Santacruz G.A., Jiménez-Bremont J.F. (2008). Salt stress increases the expression of P5CS gene and induces proline accumulation in cactus pear. Plant Physiol. Biochem..

[36] Iqbal N., Umar S., Khan N.A., Khan M.I.R. (2014). A new perspective of phytohormones in salinity tolerance: Regulation of proline metabolism. Environ. Exp. Bot..

[37] Tester M., Davenport R. (2003). Na^+^ tolerance and Na^+^ transport in higher plants. Ann. Bot..

[38] Saito K., Matsuda F. (2010). Metabolomics for functional genomics, systems biology, and biotechnology. Annu. Rev. Plant Biol..

[39] Gong Q., Li P., Ma S., Indu Rupassara S., Bohnert H.J. (2005). Salinity stress adaptation competence in the extremophile *Thellungiella halophila* in comparison with its relative *Arabidopsis thaliana*. Plant J..

[40] Van Zelm E., Zhang Y., Testerink C. (2020). Salt Tolerance Mechanisms of Plants. Annu. Rev. Plant Biol..

[41] Zandalinas S.I., Balfagón D., Gómez-Cadenas A., Mittler R. (2022). Plant responses to climate change: Metabolic changes under combined abiotic stresses. J. Exp. Bot..

[42] Tian J., Pang Y., Yuan W., Peng J., Zhao Z. (2022). Growth and nitrogen metabolism in *Sophora japonica* (L.) as affected by salinity under different nitrogen forms. Plant Sci..

[43] Zeghichi S., Kallithraka S., Simopoulos A.P. (2003). Nutritional composition of Molokhia (*Corchorus olitorius*) and stamnagathi (*Cichorium spinosum*). World Rev. Nutr. Diet..

[44] Petropoulos S.A., Fernandes A., Ntatsi G., Levizou E., Barros L., Ferreira I.C.F.R. (2016). Nutritional profile and chemical composition of *Cichorium spinosum* ecotypes. LWT-Food Sci. Technol..

[45] Petropoulos S.A., Levizou E., Ntatsi G., Fernandes A., Petrotos K., Akoumianakis K., Barros L., Ferreira I.C.F.R. (2017). Salinity effect on nutritional value, chemical composition and bioactive compounds of *Cichorium spinosum* L.. Food Chem..

[46] Chatzigianni M., Alkhaled B., Livieratos I., Stamatakis A., Ntatsi G., Savvas D. (2018). Impact of nitrogen source and supply level on growth, yield and nutritional value of two contrasting ecotypes of *Cichorium spinosum* L. grown hydroponically. J. Sci. Food Agric..

[47] Chatzigianni M., Ntatsi G., Theodorou M., Stamatakis A., Livieratos I., Rouphael Y., Savvas D. (2019). Functional Quality, Mineral Composition and Biomass Production in Hydroponic Spiny Chicory (*Cichorium spinosum* L.) Are Modulated Interactively by Ecotype, Salinity and Nitrogen Supply. Front. Plant Sci..

[48] Savvas D., Adamidis C. (1999). Automated management of nutrient solutions based on target electrical conductivity, pH, and nutrient concentration ratios. J. Plant Nutr..

[49] Papadopoulou E.A., Giaki K., Angelis A., Skaltsounis A.L., Aliferis K.A. (2022). A metabolomic approach to assess the toxicity of the olive tree endophyte *Bacillus* sp. PTA13 lipopeptides to the aquatic macrophyte *Lemna minor* L.. Toxics.

[50] Kostopoulou S., Ntatsi G., Arapis G., Aliferis K.A. (2020). Assessment of the effects of metribuzin, glyphosate, and their mixtures on the metabolism of the model plant *Lemna minor* L. applying metabolomics. Chemosphere.

[51] Tsugawa H., Cajka T., Kind T., Ma Y., Higgins B., Ikeda K., Kanazawa M., VanderGheynst J., Fiehn O., Arita M. (2015). MS-DIAL: Data-independent MS/MS deconvolution for comprehensive metabolome analysis. Nat. Methods.

[52] Babicki S., Arndt D., Marcu A., Liang Y., Grant J.R., Maciejewski A., Wishart D.S. (2016). Heatmapper: Web-enabled heat mapping for all. Nucleic Acids Res..

[53] Bardou P., Mariette J., Escudié F., Djemiel C., Klopp C. (2014). jvenn: An interactive Venn diagram viewer. BMC Bioinform..

[54] Zhu J.K. (2003). Regulation of ion homeostasis under salt stress. Curr. Opin. Plant Biol..

[55] Mansour M.M.F. (2000). Nitrogen containing compounds and adaptation of plants to salinity stress. Biol. Plant..

[56] Chen W., Meng C., Ji J., Li M.H., Zhang X., Wu Y., Xie T., Du C., Sun J., Jiang Z. (2020). Exogenous GABA promotes adaptation and growth by altering the carbon and nitrogen metabolic flux in poplar seedlings under low nitrogen conditions. Tree Physiol..

[57] Li L., Dou N., Zhang H., Wu C. (2021). The versatile GABA in plants. Plant Signal Behav..

[58] Silveira J.A.G., Viégas R.D.A., da Rocha I.M.A., Moreira A.C.D.O.M., Moreira R.D.A., Oliveira J.T.A. (2003). Proline accumulation and glutamine synthetase activity are increased by salt-induced proteolysis in cashew leaves. J. Plant Physiol..

[59] Rai S.P., Luthra R., Kumar S. (2003). Salt-tolerant mutants in glycophytic salinity response (GSR) genes in *Catharanthus roseus*. Theor. Appl. Genet..

[60] Hayat S., Hayat Q., Alyemeni M., Wani A., Pichtel Ahmad A. (2012). Role of proline under changing environments. Plant Signal. Behav..

[61] Bartels D., Sunkar R. (2005). Drought and salt tolerance in plants. Crit. Rev. Plant Sci..

[62] Rajput V.D., Harish, Singh R.K., Verma K.K., Sharma L., Quiroz-Figueroa F.R., Meena M., Gour V.S., Minkina T., Sushkova S. (2021). Recent Developments in Enzymatic Antioxidant Defence Mechanism in Plants with Special Reference to Abiotic Stress. Biology.

[63] Widodo J.H.P., Newbigin E., Tester M., Bacic A., Roessner U. (2009). Metabolic responses to salt stress of barley (*Hordeum vulgare* L.) cultivars, Sahara and Clipper, which differ in salinity tolerance. J. Exp. Bot..

[64] Wu D., Cai S., Chen M., Ye L., Chen Z., Zhang H., Dai F., Wu F., Zhang G. (2013). Tissue metabolic responses to salt stress in wild and cultivated barley. PLoS ONE.

[65] Cuin T.A., Shabala S. (2005). Exogenously supplied compatible solutes rapidly ameliorate NaCl-induced potassium efflux from barley roots. Plant Cell Physiol..

[66] Hessini K. (2022). Nitrogen form differently modulates growth, metabolite profile, and antioxidant and nitrogen metabolism activities in roots of *Spartina alterniflora* in response to increasing salinity. Plant Physiol. Biochem..

[67] Ahanger M.A., Qin C., Begum N., Maodong Q., Dong X.X., El-Esawi M., El-Sheikh M.A., Alatar A.A., Zhang L. (2019). Nitrogen availability prevents oxidative effects of salinity on wheat growth and photosynthesis by up-regulating the antioxidants and osmolytes metabolism, and secondary metabolite accumulation. BMC Plant Biol..

[68] Behr J.H., Bouchereau A., Berardocco S., Seal C.E., Flowers T.J., Zörb C. (2017). Metabolic and physiological adjustment of *Suaeda maritima* to combined salinity and hypoxia. Ann. Bot..

[69] Zhang J., Yang D., Li M., Shi L. (2016). Metabolic profiles reveal changes in wild and cultivated soybean seedling leaves under salt stress. PLoS ONE.

[70] Hildebrandt T.M., Nesi A.N., Araújo W.L., Braun H.P. (2015). Amino acid catabolism in plants. Mol. Plant.

[71] Gill S.S., Tuteja N. (2010). Reactive oxygen species and antioxidant machinery in abiotic stress tolerance in crop plants. Plant Physiol. Biochem..

[72] Kiani-Pouya A., Roessner U., Jayasinghe N.S., Lutz A., Rupasinghe T., Bazihizina N., Bohm J., Alharbi S., Hedrich R., Shabala S. (2017). Epidermal bladder cells confer salinity stress tolerance in the halophyte quinoa and *Atriplex* species. Plant. Cell Environ..

[73] Kumari A., Parida A.K. (2018). Metabolomics and network analysis reveal the potential metabolites and biological pathways involved in salinity tolerance of the halophyte *Salvadora persica*. Environ. Exp. Bot..

[74] Anjum S., Xie X., Wang L. (2011). Morphological, physiological and biochemical responses of plants to drought stress. Afr. J. Agric. Res..

[75] Wani A., Ahmad A., Hayat S., Fariduddin Q. (2013). Salt-induced modulation in growth, photosynthesis and antioxidant system in two varieties of *Brassica juncea*. Saudi J. Biol. Sci..

[76] Hu M., Shi Z., Zhang Z., Zhang Y., Li H. (2012). Effects of exogenous glucose on seed germination and antioxidant capacity in wheat seedlings under salt stress. Plant Growth Regul..

[77] Almodares A., Hadi M., Dosti B. (2008). The effects of salt stress on growth parameters and carbohydrates contents in sweet sorghum. Res. J. Environ. Sci..

[78] Rosa M., Prado C., Podazza G., Interdonato R., Gonzalez J.A., Hilal M., Prado F.E. (2009). Soluble sugars- metabolism, sensing and abiotic stress. Plant Signal. Behav..

[79] Gibson S. (2005). Control of plant development and gene expression by sugar signaling. Curr. Opin. Plant Biol..

[80] Pattanagul W., Thitisaksakul M. (2008). Effect of salinity stress on growth and carbohydrate metabolism in three rice (*Oryza sativa* L.) cultivars differing in salinity tolerance. Indian J. Exp. Biol..

[81] Cuoee I., Sulmon C., Gouesbet G., Amrani A. (2006). Involvement of soluble sugars in reactive oxygen species balance and responses to oxidative stress in plants. J. Exp. Bot..

[82] Nemati I., Moradi F., Gholizadeh S., Esmaeili M., Bihamta M. (2011). The effect of salinity stress on ions and soluble sugars distribution in leaves, leaf sheaths and roots of rice (*Oryza sativa* L.) seedlings. Plant Soil Environ..

[83] Llanes A., Arbona V., Gomez-Cadenas A., Luna V. (2016). Metabolomic profiling of the halophyte *Prosopis strombulifera* shows sodium salt- specific response. Plant Physiol. Biochem..

[84] Hafiz Che-Othman M.H., Jacoby R.P., Millar A.H., Taylor N.L. (2020). Wheat mitochondrial respiration shifts from the tricarboxylic acid cycle to the GABA shunt under salt stress. New Phytol..

[85] Kumari A., Das P., Parida A.K., Agarwal P.K. (2015). Proteomics, metabolomics and ionomics perspectives of salinity tolerance in halophytes. Front. Plant Sci..

[86] Yancey P.H. (2005). Organic osmolytes as compatible, metabolic and counteracting cytoprotectants in high osmolarity and other stresses. J. Exp. Biol..

[87] Conde A., Chaves M.M., Gerós H. (2011). Membrane transport, sensing and signaling in plant adaptation to environmental stress. Plant Cell Physiol..

[88] Sengupta S., Mukherjee S., Goswami L. (2012). Manipulation of inositol metabolism for improved plant survival under stress: A network engineering approach. J. Plant Biochem. Biotechnol..

[89] Al-Mushhin A.A.M., Qari S.H., Fakhr M.A., Alnusairi G.S.H., Alnusaire T.S., ALrashidi A.A., Latef A.A.H.A., Ali O.M., Khan A.A., Soliman M.H. (2021). Exogenous Myo-Inositol Alleviates Salt Stress by Enhancing Antioxidants and Membrane Stability via the Upregulation of Stress Responsive Genes in *Chenopodium quinoa* L.. Plants.

[90] Richter J.A., Erban A., Kopka J., Zörb C. (2015). Metabolic contribution to salt stress in two maize hybrids with contrasting resistance. Plant Sci..

[91] Liu X., Wu H., Ji C., Wei L., Zhao J., Yu J. (2013). An integrated proteomic and metabolomic study on the chronic effects of mercury in Suaeda salsa under an environmentally relevant salinity. PLoS ONE.

[92] Cooke D.T., Burden R.S. (1990). Lipid modulation of plasma membrane-bound ATPases. Physiol. Plant.

[93] Wu J., Seliskar D.M., Gallagher J.L. (1998). Stress tolerance in the marsh plant *Spartina patens*: Impact of NaCl on growth and root plasma membrane lipid composition. Physiol. Plant..

[94] Elkahoui S., Smaouri A., Zarrouk M., Ghrir R., Limam F. (2004). Salt-induced lipid changes in *Catharanthus roseus* cultured cell suspensions. Phytochemistry.

[95] Liu S.S., Wang W.Q., Li M., Wan S.B., Sui N. (2017). Antioxidants and unsaturated fatty acids are involved in salt tolerance in peanut. Acta Physiol. Plant..

[96] Sonnewald U., Brauer M., Schaewen A., Stitt M., Willmitzer L. (1991). Transgenic tobacco plants expressing yeast-derived invertase in either the cytosol, vacuole or apoplast: A powerful tool for studying sucrose metabolism and sink/source interactions. Plant J..

[97] Zolman B.K., Silva I.D., Bartel B. (2001). The Arabidopsis pxa1 mutant is defective in an ATP-binding cassette transporter-like protein required for peroxisomal fatty acid beta-oxidation. Plant Physiol..

[98] Rinaldi M.A., Patel A.B., Park J., Lee K., Strader L.C. (2016). Arabidopsis thaliana mutants with altered seedling peroxisome size or distribution reveal genes important for peroxisomal metabolism. Genetics.

