# Supplementary figures and images for: Combined Effect of Salt Stress and Nitrogen Level on the Primary Metabolism of Two Contrasting Hydroponically Grown Cichorium spinosum L. Ecotypes
Source: Biomolecules. 2023 Mar 28;13(4):607. doi: 10.3390/biom13040607 (PMC10136004; doi:10.3390/biom13040607)

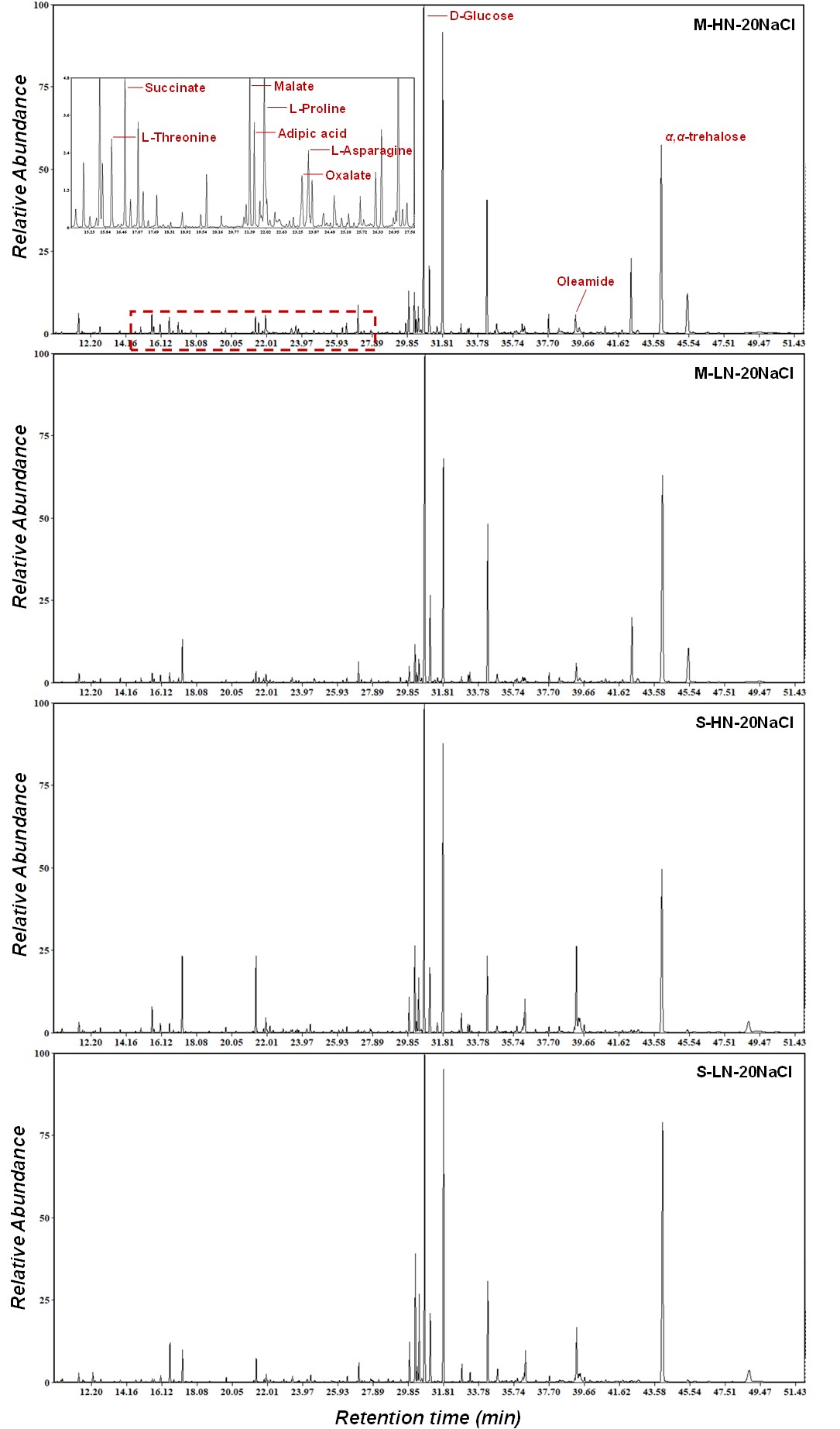

Supplement: Supplementary file 1 [file biomolecules-13-00607-s001.zip › Figure S1.jpg]

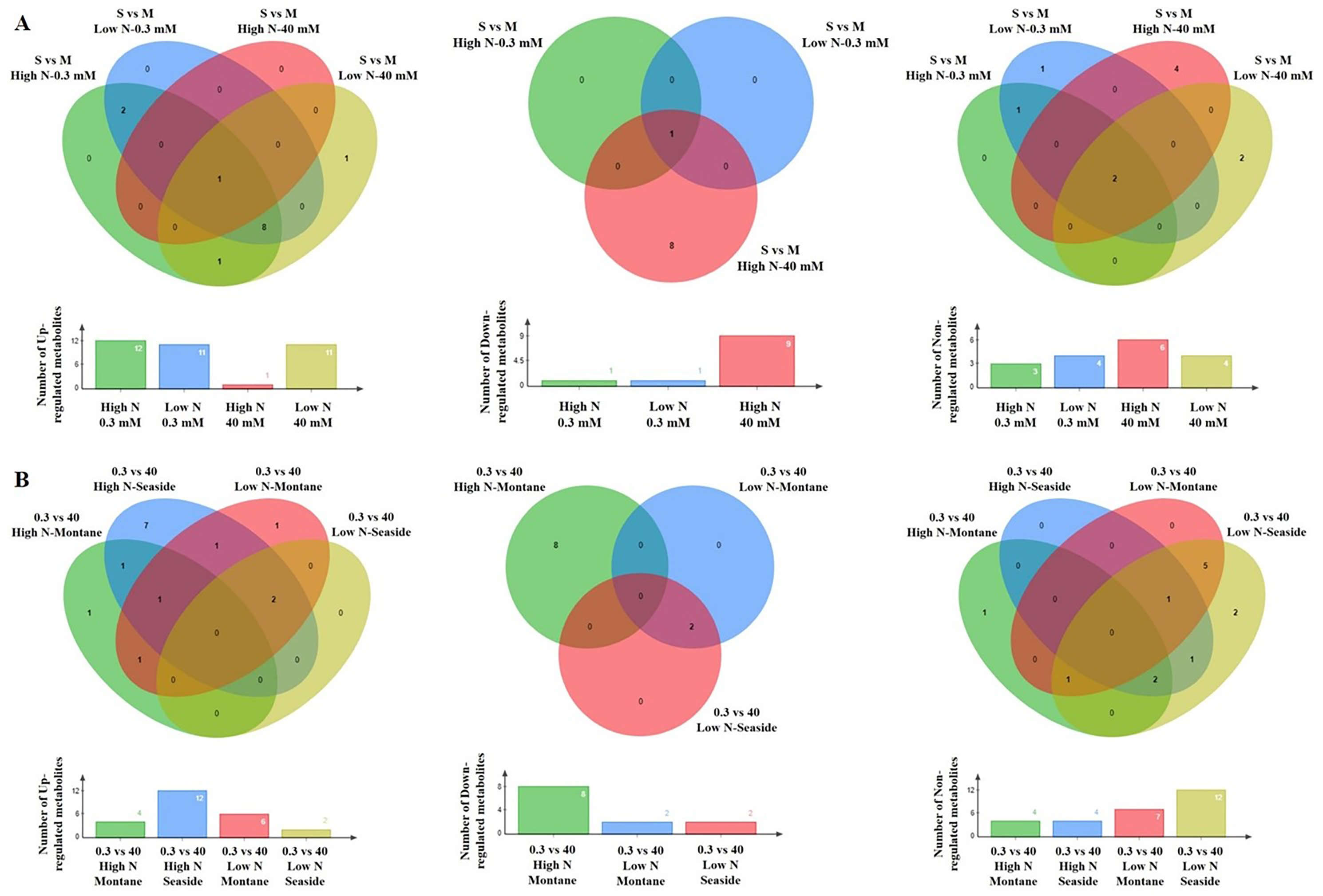

Supplement: Supplementary file 1 [file biomolecules-13-00607-s001.zip › Figure S2.jpg]

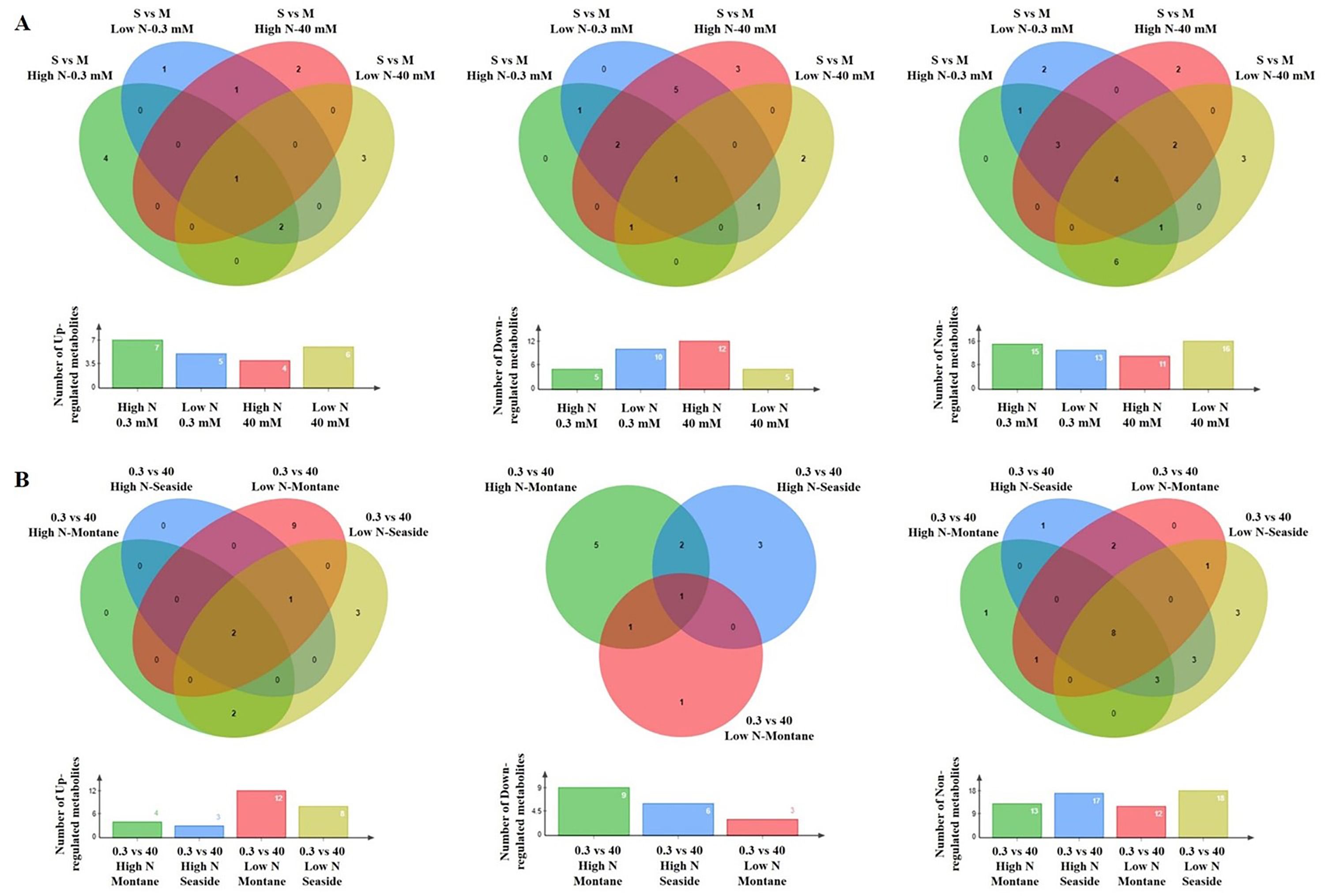

Supplement: Supplementary file 1 [file biomolecules-13-00607-s001.zip › Figure S3.jpg]

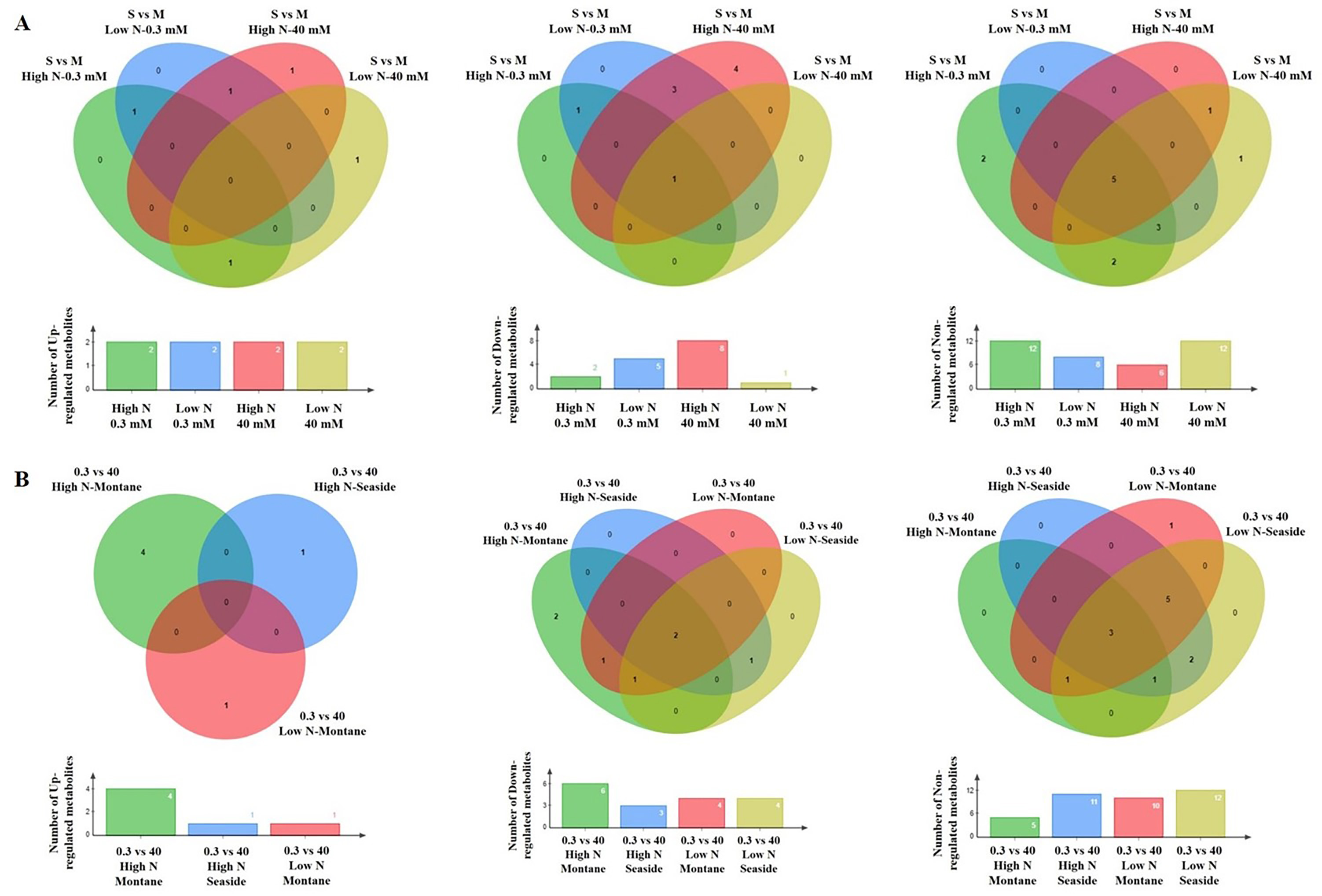

Supplement: Supplementary file 1 [file biomolecules-13-00607-s001.zip › Figure S4.jpg]

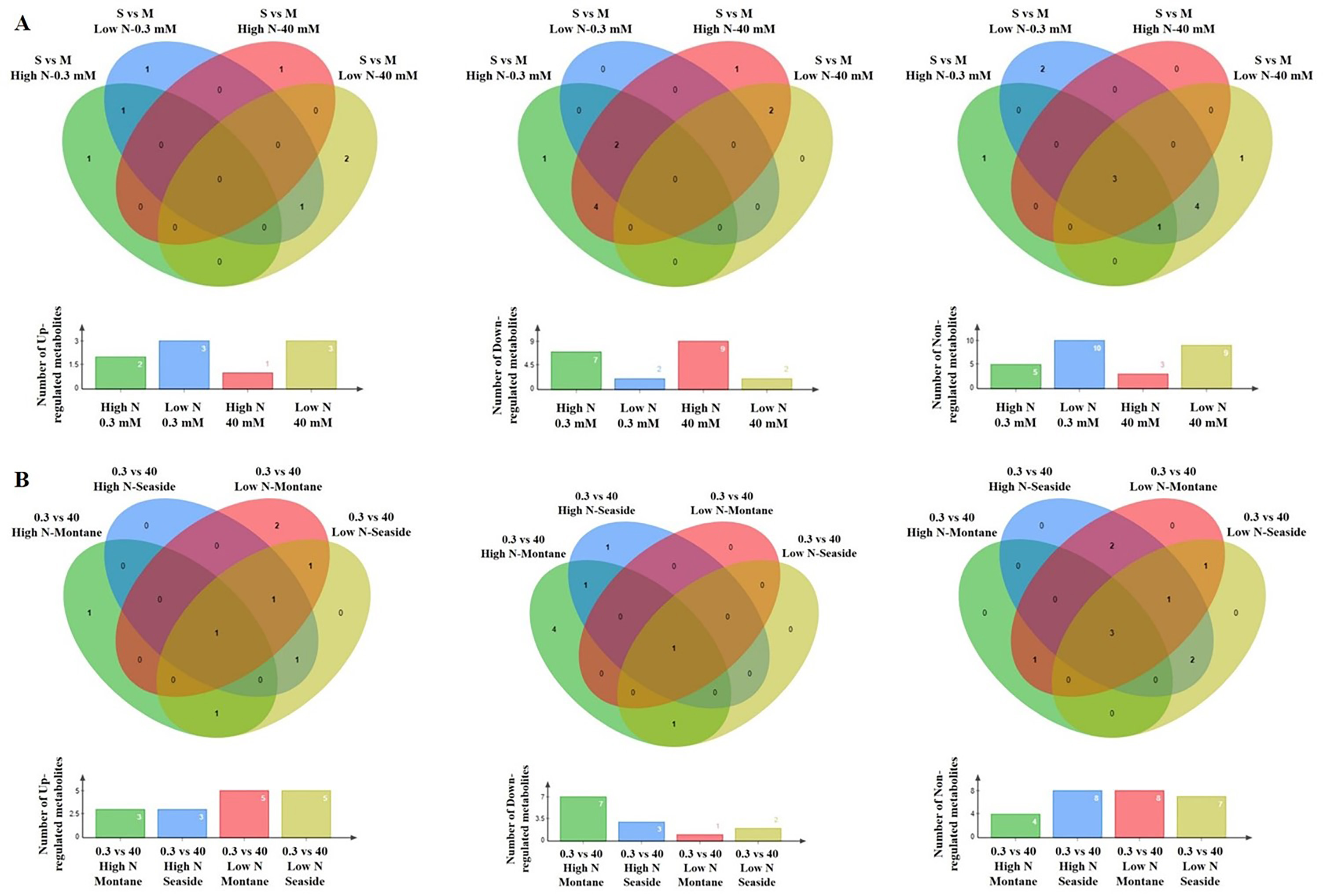

Supplement: Supplementary file 1 [file biomolecules-13-00607-s001.zip › Figure S5.jpg]
